# Liquid and Solid Self-Emulsifying Drug Delivery Systems (SEDDs) as Carriers for the Oral Delivery of Azithromycin: Optimization, In Vitro Characterization and Stability Assessment

**DOI:** 10.3390/pharmaceutics12111052

**Published:** 2020-11-04

**Authors:** Reem Abou Assi, Ibrahim M. Abdulbaqi, Toh Seok Ming, Chan Siok Yee, Habibah A. Wahab, Shaik Mohammed Asif, Yusrida Darwis

**Affiliations:** 1 The Discipline of Pharmaceutical Technology, School of Pharmaceutical Sciences, Universiti Sains Malaysia, Penang 11800, Malaysia; drmeer1@gmail.com (R.A.A.); ibrahimm.abdulbaqi@gmail.com (I.M.A.); smtoh@usm.my (T.S.M.); asif81ind@gmail.com (S.M.A.); 2 The Discipline of Pharmaceutical Technology, College of Pharmacy, Al-Kitab University, Altun kupri, Kirkuk 36001, Iraq; 3 Pharma Research, Wockhardt Research Center, Aurangabad 431002, India

**Keywords:** liquid SEDDs, solid SEDDs, drug delivery, self-emulsifying, cytotoxicity, MTT assay, tight junctions, Caco-2 cell, stability

## Abstract

Azithromycin (AZM) is a macrolide antibiotic used for the treatment of various bacterial infections. The drug is known to have low oral bioavailability (37%) which may be attributed to its relatively high molecular weight, low solubility, dissolution rate, and incomplete intestinal absorption. To overcome these drawbacks, liquid (L) and solid (S) self-emulsifying drug delivery systems (SEDDs) of AZM were developed and optimized. Eight different pseudo-ternary diagrams were constructed based on the drug solubility and the emulsification studies in various SEDDs excipients at different surfactant to co-surfactant (Smix) ratios. Droplet size (DS) < 150 nm, dispersity (*Đ*) ≤ 0.7, and transmittance (T)% > 85 in three diluents of distilled water (DW), 0.1 mM HCl, and simulated intestinal fluids (SIF) were considered as the selection criteria. The final formulations of L-SEDDs (L-F1_(H)_), and S-SEDDs (S-F1_(H)_) were able to meet the selection requirements. Both formulations were proven to be cytocompatible and able to open up the cellular epithelial tight junctions (TJ). The drug dissolution studies showed that after 5 min > 90% and 52.22% of the AZM was released from liquid and solid SEDDs formulations in DW, respectively, compared to 11.27% of the pure AZM, suggesting the developed SEDDs may enhance the oral delivery of the drug. The formulations were stable at refrigerator storage conditions.

## 1. Introduction

Oral drug delivery is the most widely used and the common route of drug administration because it is convenient, economical, comfortable, and requires no special training for use [1,2]. However, despite these advantages, problems such as poor solubility, low dissolution rates, and limited drug diffusion through the paracellular pathways before eventually entering the systemic circulation making it challenging for many drugs to reach the therapeutic levels via this route [3,4].

Azithromycin (AZM) is a semisynthetic 15-membered macrolide antibiotic [5], with a lipophilic nature (log P = 4), (pKa = 8.74), and a molecular weight of 749 g/mol [6,7]. The drug, which is included in the model list of essential medicines on the World Health Organization website [7], is classified as the first azalide subclass among its family members [8,9] with a superior antibacterial activity in the market for the last three decades. This makes AZM the drug of choice for the treatment of various gastrointestinal, respiratory, and genitourinary infections. Recently it has gained increased popularity owing to its suggested important role in improving the ability of hydroxychloroquine to eradicate the global outbreak of COVID-19 virus at clinical levels [10]. However, the oral intake of AZM is associated with a relatively low bioavailability of 37%. The exact reasons for this low oral bioavailability are not specified. However, many reports are suggesting that it might be attributed to different factors such as the low aqueous solubility (AZM is practically insoluble in water) which may lead to erratic dissolution rates [11,12,13,14,15], the drug relatively high molecular weight, the low stability at the acidic gastric pH (AZM have a high potential of decomposition in acidic medium) [16], and the incomplete gastrointestinal tract (GIT) absorption [17]. Pharmacokinetically, most poorly water-soluble drugs have low bioavailability. Furthermore, AZM is recognized to be a substrate for the P-glycoprotein (P-gp) efflux transporters, which can potentially restrict its transcellular diffusion and permeability [18,19,20]. To the best of our knowledge, cellular studies on the paracellular permeability of AZM and its nanoformulation(s) on colon adenocarcinoma human cells (Caco-2) line are scanty. Nevertheless, some reports are suggesting that AZM may increase the transepithelial electrical resistance (TEER) values when studied in certain cell lines such as human airway epithelial cell lines [21,22], which could be linked to changing the processing of tight junction proteins [22,23]. This may lead to a negative impact on AZM paracellular transport and permeability. All these drawbacks led to higher oral AZM dosing regimens and longer times of treatment that, in turn, intensified the associated GIT side effects, including diarrhea, nausea, and abdominal pain. The only other available dosage form of AZM is the intravenous infusion, which is also associated with severe adverse effects, including pain at the injection site and local inflammation [24].

Increasing the solubility and dissolution rates of poorly water-soluble drugs are of the most challenging tasks in drug development nowadays for enhancing their oral bioavailability. Different strategies and techniques were employed for this purpose such as complexation, chemical modification, solid dispersions, and the use of nanocarriers and drug delivery systems. Solid dispersions [11,15,25], nanosuspensions [26], and niosomes [27] of AZM were employed to enhance its solubility and dissolution rates.

Lipid-based carriers have been used successfully to enhance the oral delivery of various drugs. These formulations are generally classified into four types (I, II, III, and IV) [28,29]. In particular, type III carriers, which are also known as self-emulsifying drug delivery systems (SEDDs), have been driving a profound interest by the pharmaceutical researchers and industries for their efficacy in enhancing oral delivery of various therapeutic agents of different physiochemical properties [30,31,32,33,34]. SEDDs are used as effective tools to enhance the GIT absorption and oral bioavailability of poorly water-soluble drugs by significantly increasing their solubility and improving their dissolution behavior [35]. Furthermore, SEDDs are also existing as liquid (L-SEDDs) and solid (S-SEDDs) formulations. The S-SEDDs are suggested to provide better stability, reproducibility, and patient compliance, in addition to ease of process control [36].

The present study aimed to develop L-SEDDs and S-SEDDs formulations for potentially enhancing the oral delivery of AZM by increasing its solubility and dissolution rates using excipients that are able to solubilize the highest amounts of the drug and at the same time have a previously reported capacity to loosen the intestinal TJ. In this work, AZM-loaded L-SEDDs were prepared and characterized. The optimized liquid SEEDs were converted to solid SEDDs using various solidifying agents. All the prepared AZM-loaded liquid and solid SEEDs formulations were characterized in terms of droplet size (DS), dispersity (*Đ*), zeta potential (ZP), maximum drug content (DC) which is also called solubilization capacity, and in vitro release behaviors. The cytotoxicity and the potential abilities of the optimized liquid and solid formulations to open the epithelial TJ by reducing the trans-epithelial electrical resistance (TEER) for better drug permeability were investigated. Furthermore, the stability of the optimized formulations was evaluated at different storage conditions.

## 2. Materials and Methods

### 2.1. Materials

Azithromycin (purity > 97.2%) was a kind gift from Wockhardt research center (Aurangabad, India). Labrasol^®^, Labrafac PG^®^, Labrafil^®^ M 1944 CS, Capryol 90^®^ and Transcutol HP^®^ were purchased from Gattefossé (Lyon, France). Span 20^®^, Span 80^®^, Tween 20^®^, Tween 80^®^, octanoic acid ≥ 99%, castor oil, potassium phosphate monobasic ≥ 99%, Trypsin-EDTA solution 1× (0.25% trypsin, 0.02% EDTA), dimethyl sulfoxide ≥ 99.5% (DMSO), thiazolyl blue tetrazolium bromide 98% (MTT) and Corning^®^ transwell polycarbonate membrane cell culture inserts 24 well plates were obtained from Sigma-Aldrich (St. Louis, MO, USA). Cremophor 40^®^, Cremophor EL^®^ were bought from BASF (Ludwigshafen, Germany). Pureco^®^ 76 was purchased from Abitec corporation (Janesville, WIS, USA). Aerosil 200^®^ ≥ 99.8% was obtained from Evonik Inc. (Essen, Germany). Calcium carbonate, mannitol, and sodium hydroxide pellets were brought from R & M chemicals Ltd. (Essex, UK). HMS lactose was ordered from B.V. Hollandsche Melksuikerfabriek (Uitgeest, Holland). The medium used for the cell culture was Gibco^®^ Dulbecco’s modified eagle’s medium (DMEM (1×) + GlutaMAXTM-1), penicillin-streptomycin solution (Pen-Strep) (which contains 10,000 units of penicillin, and 10 mg of streptomycin), and Gibco^®^ fetal bovine serum (FBS) (New Zealand origin), along with Gibco^®^ Dulbecco’s phosphate-buffered saline (1×) (DPBS), and hydrochloric acid (HCl) were bought from ThermoFisher scientific (Waltham, MA, USA). Caco-2 cells line was obtained from the American Type Culture Collection (ATCC) (Manassas, VA, USA). Palm oil was purchased from a local Malaysian market. All other used organic solvents or chemicals were either of analytical or HPLC grades.

### 2.2. Methods

#### 2.2.1. HPLC Analysis

AZM solubility, drug content, and the in vitro release studies were determined using a previously validated HPLC method which was specially developed for these purposes [37]. In brief, the separation was done using Hypersil GOLD C-18 analytical column packed with deactivated silica (250 mm × 4.6 mm ID × 5 µm) kept at 60 °C. A mixture of ammonium acetate solution (30 mmol/L, pH = 6.8) and acetonitrile at the ratio of (18:82, *v*/*v*) was used as the mobile phase. The UV detection was done at 210 nm. Samples were eluted isocratically at a flow rate of 0.7 mL/min. The theoretical plate (N > 1500), tailing factor (T ≤ 1.5), and resolution (Rs > 3) were as per the United States Pharmacopeia (USP) [5]. The linearity was observed over the concentration range of 5–200 μg/mL (R^2^ > 0.9999). The limit of detection (LOD) and limit of quantification (LOQ) were 0.476 µg/mL and 1.443 µg/mL, respectively. The developed method was statistically confirmed to be accurate, precise, and reproducible.

#### 2.2.2. Preparation and Characterization of Blank Liquid SEDDs

- Excipients selection and screening

Excipients selection was mainly based on their ability to solubilize the highest amounts of the AZM. The impact of lipids classes as long carbon chain triglycerides (LCT) and medium carbon chain triglycerides (MCT) was observed, as such differences in carbon chain lengths were reported to have an influence on drug solubilization potential and the ability to facilitate the emulsification process [38,39]. Extra attention was given to include excipients with previously reported abilities to loosen the cellular TJ for the possibility of enhancing the drug paracellular route diffusion. Besides, certain compounds were reported to have other cellular activities (such as P-gp inhibition) along with the TJ loosening were also investigated. Other excipients which have not been reported for owning any such cellular activity were also included for their potentials in enhancing AZM solubility, as illustrated in Table 1.

- Solubility study

The solubilization capacities of various excipients were investigated using the equilibrium method to ensure the selection of excipients that can solubilize the maximum amount of AZM. In brief, an excess amount of AZM (300 mg) was weighed in multiple 5 mL screw-capped bottles, then 3 mL of the different oils, surfactants, or co-surfactants were added separately into each bottle and vortexed for 2 min using ZX3 vortex mixer (VELP Scientifica, Italy). The filled bottles were then placed in a reciprocating shaker bath (Braun, Melsungen, Germany) at 500 oscillations/min for 72 h and maintained at 37 ± 2 °C. The bottles were centrifuged at 3000 rpm for 15 min to remove the undissolved AZM, and the resulted supernatant layer was filtered through a 0.45 µm syringe driven filter (Whatman, USA). Drug concentration in each vehicle was determined by HPLC.

- Emulsification studies

Based on the results of the AZM solubility study, the selected surfactants and co-surfactants were further screened for their emulsification abilities in the selected oils. Various surfactant to co-surfactant (Smix) ratios of (1:1), (2:1), (3:1), (4:1), (5:1), (6:1) and (1:2) (*v*/*v*) were mixed in separate vials and vortexed for few seconds until homogenous mixtures were obtained. A fixed oil ratio of 10% was added to each Smix ratio (90%); the mixtures were then gently vortexed and allowed to equilibrate at a reciprocating shaker bath for 2 h at room temperature. The produced mixtures had a range of hydrophilic–lipophilic balance (HLB) values within the range of 6.55 to 14.22. Although the characterization of SEDDs is mainly conducted using either distilled or deionized water as the dispersing medium [34,55,56,57], yet, in this work, the self-emulsification efficiency of various mixtures was evaluated on the bases of the produced droplet size (DS), dispersity (*Đ*) and transmittance percentage (T%), in three different diluents namely: distilled water (DW), 0.1 mM HCl (pH = 4), and simulated intestinal fluids (SIF) (pH = 6.8) for mimicking the oral route of administration and select the most physically stable formulation(s) [58,59]. All the used diluents were freshly prepared and filtered through a 0.45 µm nylon membrane filter before use. Each formulation underwent a standard dilution of 1:1000 ratio (10 µL sample to 10 mL diluent) then gently stirred with a magnetic stirrer and allowed to equilibrate before loading into a cuvette in a thermostatic chamber to measure DS and *Đ* through Photon Correlation Spectroscopy (PCS) using laser light scattering spectrometer Zetasizer 1000HSA (Malvern Instrument, UK). The measurement of T% was conducted at a dilution ratio of 10:1000 (100 µL sample to 10 mL diluent), then evaluated at 650 nm using a UV-Vis Spectrophotometer U-2000 (Hitachi, Japan) [60]. Only mixtures that produced DS < 150 nm, *Đ* ≤ 0.7, and T% > 85 in the three different diluents were selected for the construction of the pseudo-ternary diagram.

- Pseudo-ternary diagram construction

This step was done to identify the self-emulsifying regions of the L-SEDDs, where the total concentration of the three constituents (oil, surfactant, co-surfactant) was always 100%. A series of 19 blank L-SEDDs formulations were prepared by mixing the oil with the previously selected Smix ratios in different vials and volume ratios (1:9, 1:8.5, 1:8, 1:7.5, 1:7, 1:6.5, 1:6, 1:5.5, 1:5, 1:4.5, 1:4, 1:3.5, 1:3, 1:2.5, 1:2, 1:1.5, 1:1, 1.5:1 and 2:1). The vials were gently vortexed for 20 s and kept in the reciprocating shaker bath for two hours at room temperature. The blank L-SEDDs formulations were assessed for visual appearance and DS after preforming the employed standard dilution with filtered DW [61,62]. Only clear-transparent dispersions with DS < 150 nm were considered in the self-emulsified region of the phase diagrams for this study. The phase diagrams were plotted using CHEMIX^®^ ternary plot software (CHEMIX School Ver. 3.60, Pub. Arne Standnes, Bergen, Norway).

#### 2.2.3. Preparation and Characterization of AZM-Loaded L-SEDDs

Out of each identified self-emulsification area, three transparent formulations with DS < 150 nm were selected at three oil concentrations (low, medium, and high) for the maximum DC (or solubilization capacity) studies. There was a 5% difference between the three oil concentrations. A total of 24 L-SEDDs formulations were chosen for the DC studies. To perform the experiment, an excess amount of AZM (200 mg) was accurately weighed in multiple 5 mL screw-capped bottles, then 2 mL of each blank L-SEDDs formulation was added to each bottle. The samples were then treated as per the procedure of the AZM solubility studies, and the DC was measured. Both blank L-SEDDs and their corresponding AZM loaded L-SEDDs (AZM-L-SEDD) formulations were further characterized in terms of DS, *Đ*, T%, and zeta potential (ZP) in three diluents (DW, 0.1 mM HCl, and SIF). ZP was measured using Zetasizer nanoseries Nano-Z, (Malvern Instrument, UK). The AZM-L-SEDDs formulations with the highest AZM content, DS < 150 nm, *Đ* ≤ 0.7, T% > 85, and highest ZP value were selected for the solidification process.

#### 2.2.4. Preparation and Characterization of AZM-Loaded S-SEDDs

Various solidification methods such as adsorption to solid carriers, spray drying, freeze-drying, rotary evaporation, melt extrusion-spheronization, and melt granulation are available for the preparation of S-SEDDs [63,64]. In this work, adsorption to solid carriers’ method was adopted, as the technique is simple, which involves the addition of the L-SEDDs to the selected carriers with suitable mixing. Furthermore, the produced S-SEDDs by this method are stable and freely flowing [65,66]. Various water-soluble (mannitol and lactose) and water-insoluble (calcium carbonate and Aerosil 200^®^) solidifying agents were screened to select the suitable one for the conversion of the optimized blank L-SEDDs and AZM-L-SEDDs into blank solid SEDDs (S-SEDDs) and AZM loaded solid SEDDs (AZM-S-SEDDs), respectively.

At first, the adsorption capacity (expressed as a weight ratio of L-SEDDs: solidifying agent) of each solidifying agent was studied through a drop-wise addition and mixing of a fixed portion of blank L-SEDDs (0.5 mL~535.6 mg) with an equivalent portion(s) of the solidifying agent (~535.6 mg) in a porcelain mortar until a non-sticky solid powder was produced. The mixing was done using a glass rod after each addition to ensure uniform distribution of the formulation. The obtained S-SEDDs were left for 24 h at room temperature to dry before further characterization for their DS, *Đ*, and ZP [67,68,69]. For DS and *Đ* measurements, 10 mg of the prepared S-SEDDs were dispersed in 10 mL of filtered diluent (DW, 0.1 mM HCl, or SIF). The S-SEDDs formulations solidified with a water-soluble solidifying agent were gently stirred with a magnetic stirrer for 10 min, then allowed to equilibrate and loaded into a cuvette in a thermostatic chamber. While those S-SEDDs solidified with a water-insoluble solidifying agent, the dispersions were first centrifuged using MiniSpin^®^ plus Eppendorf centrifuge (Eppendorf Ag, Hamburg, Germany) at 8000 rpm for 10 min to remove the water-insoluble solids then the samples were loaded into a cuvette in a thermostatic chamber [70].

The solidifying agent that produced S-SEDDs formulation with the lowest DS, *Đ*, and highest ZP was selected for solidifying AZM-L-SEDDs. Formulations were further examined for their DS, *Đ*, and ZP in the previously mentioned three diluents. AZM concentration in AZM-S-SEDDs formulation was quantified by dissolving the solid formulation (1.51 g) in 10 mL methanol and stirring it with a magnetic stirrer for 30 min. The solution was then sonicated in an ultrasonic bath (Branson 5510, Las Animas, CA, USA) for 2 min. Suitable aliquots were taken and diluted with the diluting solution and filtered with a 0.2 µm pore size nylon filter, then injected into HPLC.

#### 2.2.5. Transmission Electron Microscope (TEM)

The morphology and shape of the final selected SEDD formulations in their liquid and solid forms were studied by TEM. A drop of the dispersed liquid or solid formulation in DW (as per the dispersing procedures for DS and *Đ* measurements) was placed onto 400 mesh carbon-coated copper grid, air dried, then negatively stained with 2% phosphotungstic acid for 5 min at room temperature. After that, the excess sample was removed using a filter paper and allowed to dry before observation. Images were taken under the transmission electron microscope TEM (LIBRA 120, Carl Zeiss, Oberkochen, Germany).

#### 2.2.6. Cell Culture Studies

- Cell stock preparation

Caco-2 cells with passage numbers of 25 and 26 were grown and maintained on culture flask in DMEM supplemented with 10% *v/v* of FBS and 1% *v/v* of 1% Pen-Strep (named as the complete medium) and were maintained at 37 °C in an atmosphere of 5% CO_2_ and 90% relative humidity in a Binder^®^ constant climate chamber (Tuttlingen, Germany) [71]. Once 80% of the flask surface is covered by cell monolayer, cells were split and washed twice with DPBS, then the trypsin-EDTA solution was added and gently swirled, ensuring the complete covering of trypsin to all the cells. The flask was then incubated for 10 min in an atmosphere of 5% CO_2_ and 90% relative humidity. Cells stock was prepared by aspirating out the trypsin-EDTA, and adding the complete medium, then centrifuging this cells stock in HERAEUS^®^ LABOFUGE 400R centrifuge ThermoFisher Scientific (Waltham, MA, USA) at 900 rpm for 5 min to create a pellet at the bottom of the centrifuge tube. The cell pellet was re-suspended in 3 mL of fresh complete medium for further use as a cell stock. All cell culture buffers and solutions were pre-warmed to 37 °C prior to contact with the cells, and all protocols involved the handling of Caco-2 cells in cultures were performed aseptically.

- Cytotoxicity assay

The cytocompatibility of the optimized blank L-SEDDS and S-SEDDs formulas was investigated using the microtiter tetrazolium assay (MTT). The assay was done according to previously described methods [72,73], with slight modifications. Proper dilutions of the cell stock with the complete medium were made to get the required seeding density of 51,000 cells/cm^2^ on Biofil^®^ tissue culture 96 well plates (Guangzhou, China).

After 24 h of seeding on the 96 well plates at 37 °C in an incubator with 5% CO_2_ and 90% relative humidity, the attached cells were washed twice with DPBS, then concentrations of blank L-SEDDS (0.25, 0.5, 1, and 2% *v*/*v*) and blank S-SEEDs (0.39, 0.77, 1.54, and 3.08% *w*/*v*) were selected for the study. The SEDDs were dispersed in DMEM solution, gently vortexed, filtered using a 0.2 µm sterile filter membrane, and added to the 96 wells plates then incubated for 4 h. DMEM alone was employed as blank. After incubation, the supernatant layer was discarded, and all cells were washed twice with DPBS. To observe cells’ vibrant reductive activity, a final concentration of 1 mg/mL of thiazolyl blue tetrazolium bromide dye solution (MTT solution) was dissolved in DPBS within a tube wrapped in aluminum foil to protect it from light, and the cells in the well plates were loaded with 100 µL of sterile and filtered MTT solution and incubated for another 4 h. Then the supernatant layers were removed, the precipitated formazan crystals were dissolved in DMSO, and the plates were gently rotated on an orbital shaker for 5 min. The color of the resulted formazan solutions in DMSO was measured at 570 nm with background subtraction at 690 nm by a Multiskan™ FC microplate photometer (Thermo Scientific, Waltham, MA, USA) with Skanit software version 3.2. The percentage of cells viability was calculated with respect to the control [74], as follows:

(1) $$Viability\;\%=\frac{Abs\;sample-Abs\;blank}{Abs\;control-Abs\;blank}*100$$

where, *Abs _sample_*: Absorbance of cells treated with formulations. *Abs _control_*: Absorbance of cells not treated with formulations. *Abs _blank_*: Absorbance of blank.

- Transepithelial electrical resistance evaluation

**Figure 1:**
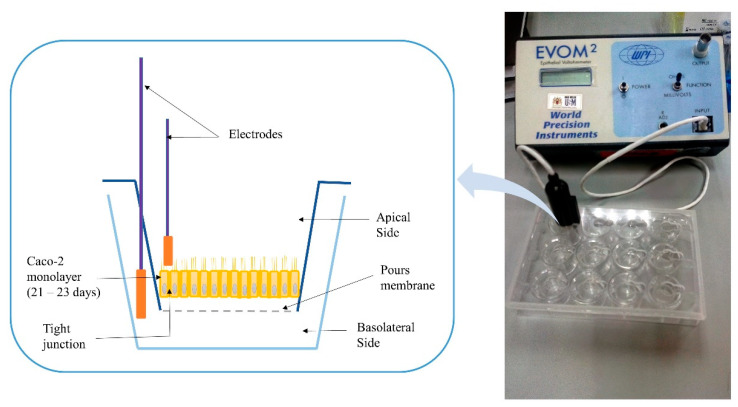
Schematic diagram for the measuring procedure of transepithelial electrical resistance (TEER).

Transepithelial electrical resistance (TEER) is a widely accepted quantitative technique to measure the integrity of tight junction dynamics in cell culture models of epithelial monolayers where the TJ proteins in the paracellular route contribute to an ohmic resistance (RTEER) in the equivalent circuit [75]. The TJ opening was investigated as per the previous protocol [76]. Cells suspension corresponding to 51,000 cells/cm^2^ was prepared from the cells’ stock and added to the apical transwell compartments of all plates. The plates were maintained at 37 °C in an incubator with 5% CO_2_ and 90% relative humidity until they formed a polarized/differentiated monolayer, which took approximately a period of 21–23 days. During that period, a regular replacement of cells medium in both chambers (every 48 h), and monolayer’s integrity monitoring were conducted by measuring the TEER using an EVOM2^®^ epithelial volt ohmmeter with STX100 electrodes (World Precision Instruments Ltd., Stevenage, UK). Figure 1 shows a schematic diagram of the measuring procedure of the TEER. The Caco-2 cell monolayer with average TEER values ≥ 300 Ω.cm^2^ indicated that cells are intact [69], and accordingly, they were used in this study. Raw data were measured in Ohm (Ω) and converted to Ω × cm^2^ based on the area of transwell plate inserts, which is equal to 0.4 cm^2^ for the used 24-well plate [77].

On the day of the experiment, the cell cultures medium was removed, the monolayers were washed with DPBS, and fresh medium was placed in both chambers; cell cultures were left for 15 min. to equilibrate before starting the measurements. The monolayers TEER values were measured in all the studied plates for the first 10 min of the test before adding any samples, including DMSO as control, DMEM as blank, pure AZM, blank and AZM incorporated L-SEDDs, as well as blank and AZM incorporated S-SEDDs formulations.

The selected studied concentration was based on the MTT assay results. The blank and AZM incorporated L-SEDDs at the concentration of 0.5%, and an equivalent concentration of blank and AZM incorporated S-SEDDs formulations, as well as pure AZM, were dispersed in DMEM. All samples were filtered using a 0.2 µm sterile filter membrane immediately before use. At the apical side, 10 µL of the medium was replaced with 10 µL of each of the seven studied solutions, and once the addition is made, TEER values were measured at different time intervals including, every minute for 30 min, and then every hour for a period of 4 h. The resistance of the monolayer was calculated using the following equation:

***R****cell layer* = ***R** sample* − ***R** blank* (2)

where, ***R*** *cell layer*: Resistance of the Caco-2 monolayer. ***R*** *sample*: Resistance reading of the studied sample. ***R*** *blank*: Resistance reading of the blank.

#### 2.2.7. The In Vitro AZM Release Studies

The in vitro release of the optimized L-SEDDs and S-SEDDs formulations was performed in comparison to the pure AZM powder using Varian^®^ 7000 USP dissolution apparatus II (Santa Clara, CA, USA), paddle method, as recommended by USFDA [78]. Three different dissolution media were employed, namely: DW, 0.1 mM HCl (pH = 4), and SIF (pH = 6.8). The SIF was prepared by mixing 250 mL of 0.2 M potassium phosphate monobasic solution with 118 mL of 0.2 M sodium hydroxide solution then diluted with DW to 1000 mL [79]. A volume of 900 mL of the selected dissolution medium was placed in each vessel of the dissolution apparatus and was kept at 37 ± 2 °C with a paddle rotating speed of 100 rpm. An amount of 100 mg of pure AZM powder, and equivalent volume and weight of AZM-loaded L-SEDDs and S-SEDDs formulations that are containing 100 mg of the drug were placed in the vessels [15,80]. From each vessel, 5 mL aliquot was drawn at selected time intervals of 5, 15, 30, 60, 90, 120, 180, and 240 min, respectively, and were filtered through 0.45 µm polytetrafluoroethylene (PTEF) filters (Titan^®^, West Springfield, MA, USA). The drawn aliquots were directly replaced with an equal volume of the same fresh dissolution media, and AZM was quantified by HPLC.

#### 2.2.8. Stability Studies

Stability studies of the optimized AZM incorporated L-SEDDS and S-SEDDS formulas were conducted for three months under three different temperatures and relative humidity conditions, namely refrigerator (4 ± 2 °C), room condition (25 ± 2 °C and 60 ± 5 relative humidity %), and humidity chamber (40 ± 2 °C and 75 ± 5 relative humidity %). The samples were analyzed for their DS, *Đ*, ZP, DC %, and the in vitro release profiles at different time intervals of 0, 0.5, 1, 2, and 3 months.

#### 2.2.9. Statistical Analysis

All experiments were carried out for at least in triplicate. To test the statistical significance, a one-way analysis of variance (ANOVA) with Tukey’s HSD (honest significant difference) tests were used. The difference in all the analyses was considered statistically significant when *p* < 0.05. All the statistical tests were done using (Minitab^®^ statistical software, version 17.2.1.0, Minitab Inc., USA).

## 3. Results and Discussion

### 3.1. Excipients Selection and Solubility Studies

**Figure 2:**
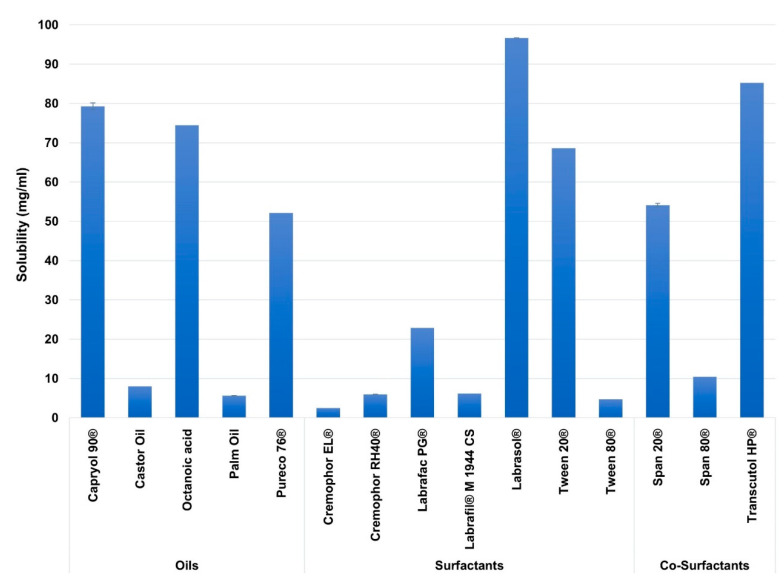
The solubility of Azithromycin (AZM) in the different screened excipients. Mean ± SD, n = 3.

In this study, excipients of different carbon chain lengths were employed for better screening of SEDDs ingredients. Figure 2 illustrates the solubility of AZM in the screened excipients.

Among the studied excipients, the highest solubility of AZM was observed in the oils of Capryol 90^®^ (80.16 ± 0.83 mg/mL), and Octanoic acid (74.5 ± 0.035 mg/mL), in the surfactants of Labrasol^®^ (97.44 ± 0.7 mg/mL), and Tween 20^®^ (69.43 ± 0.81 mg/mL) and in the co-surfactants of Transcutol^®^ HP (85.41 ± 0.27 mg/mL), and Span 20^®^ (53.48 ± 0.75 mg/mL).

Based on the literature review, many reports showed that drugs’ solubilities in various oils and surfactants might vary based on the carbon chain lengths of the oils and surfactants [81], including MCT [82,83,84], and LCT [85,86]. This attitude is probably linked to the employed drug physicochemical properties [82,87]. Among the studied excipients, AZM showed higher solubility in the MCT rather than the LCT. This may be linked to the high log *p*-value of AZM (4.02), where such drugs are shown to be more soluble in MCT than LCT [88]. Furthermore, MCT are reported to have a higher solvent capacity [89,90]. In contrast, AZM showed poor solubility in Cremophor RH40^®^ and Cremophor EL^®^ despite being medium-chain surfactants; this is likely because these surfactants are derived from edible oil (castor oil), where some lipophilic drugs reported to have very low solubility in such oils during SEDDs formulation studies [91,92,93].

### 3.2. Emulsification Efficiency Studies

Based on the solubility study results, all possible combinations of the various excipients were investigated for emulsification efficiency, as illustrated in Table 2. As more than two types of chemicals were incorporated in the SEDDs formulations, then one of the components was used at a fixed ratio (oil) [94], while others (Smix) were studied at the previously mentioned ratios in the method section. Precisely, a fixed ratio of 10% of the oil was employed as recommended to produce spontaneous L-SEDDs formulations with small DS upon dispersion [28].

As the present SEDDs formulations are designed for oral delivery, the potential effects of GIT conditions on SEDDs emulsification were considered by performing the emulsification studies in different diluents, namely DW, 0.1 mM HCl (pH = 4), and SIF (pH = 6.8). The formulations that were able to maintain their DS < 150 nm, *Đ* ≤ 0.7, and T% > 85 in all the three diluents were selected for the next steps.

The selection criteria are based on the fact that the smaller DS is associated with greater absorption, and faster release, facilitated hydrophobic drug solubilization, and quick transport from the stomach and distribution along the GIT [38,95,96,97]. Furthermore, the L-SEDDS formulations with DS < 150 nm showed higher size robustness upon dilutions in the different media in the emulsification study. On the other hand, smaller *Đ* values were required to get a better system homogeneity [98,99]. While T% > 85 was considered, as it indicates good emulsification in terms of rapid and reproducible equilibrium [100,101,102].

By performing the emulsification studies, Tween 20^®^ was shown to be superior to Labrasol^®^ in emulsifying Capryol 90^®^ or Octanoic acid in A1, A2, and B1, B2 formulations. This ability might be attributed to the higher HLB value of Tween 20^®^ (16.7) than the HLB value of Labrasol^®^ (12–14) [103,104].

Based on the discussed selection criteria, C1 and C2 formulations at the Smix ratios of (2:1), (5:1), (6:1) and (2:1), (3:1), (4:1), (5:1), (6:1), respectively were selected for further studies through the construction of pseudo-ternary diagram.

### 3.3. Construction of Pseudo-Ternary Phase Diagram

**Figure 3:**
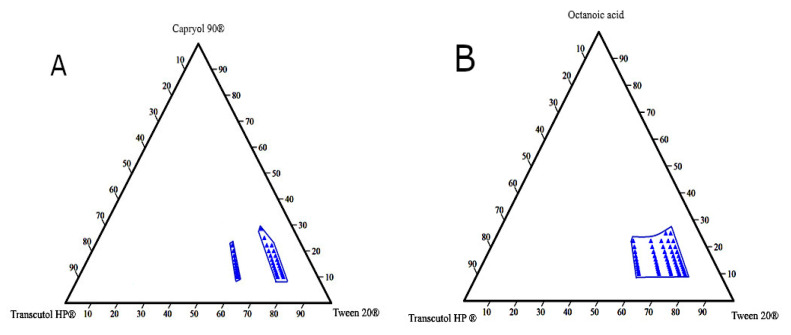
The pseudo-ternary diagram plots for the L-SEDDs formulations. (**A**) For the L-SEDDs formulation composed of Capryol 90^®^ (oil), Tween 20^®^ (surfactant), and Transcutol HP^®^ (co-surfactant) at different Smix ratios. (**B**) For the L-SEDDs formulations composed of Octanoic acid (oil), Tween 20^®^ (surfactant), and Transcutol HP^®^ (co-surfactant) at different Smix ratios.

Ternary phase diagrams are constructed to determine self-emulsification areas at which various concentrations of the excipients (oil, surfactant, and co-surfactant) would possess a transparent appearance and DS < 150 nm. These parameters were considered for selecting the optimum L-SEDDs formulations. Based on the emulsification study, eight diagrams were constructed (Figure 3).

Different blank L-SEDDs samples with three levels of oil: Smix ratios including (8:1), (5:1), and (3.5:1) that meet the set selection criteria were further investigated. These ratios represent high (H), medium (M), and low (L) concentrations on the pseudo-ternary diagram as described in Table 3. This approach was considered to avoid the use of a high concentration of surfactants (>60%), which is associated with GIT irritation when used in L-SEDDS formulations [105,106]. In the pseudo ternary phase diagram, each blue dot represents a formulation with various combination ratios of (oil: surfactant: co-surfactant) where all set criteria are met.

### 3.4. Preparation of AZM Loaded L-SEDDs

#### AZM Incorporation and Its Impact on L-SEDDs Properties

The optimal drug incorporation into the L-SEDDs formulations depends on the compatibility between the added drug and the physicochemical properties of the formulation. The results of AZM incorporation and its related impacts on the L-SEDDs characteristics are shown in Table 4 and Table 5.

The results of exploring DC or solubilization capacity of various formulations showed that it is directly proportional to the oil concentration increment where L-F1_(L)_, L-F1_(M)_, and L-F1_(H)_ formulations were found to have a DC of 12.3 ± 0.05, 32.23 ± 0.07, and 60.42 ± 0.4 mg/mL, respectively. While the DC of L-F2_(L)_, L-F2_(M)_, and L-F2_(H)_ formulations were 18.68 ± 0.16, 29.97 ± 0.14 and 46.9 ± 0.67 mg/mL, respectively. L-F1_(H)_ and L-F2_(H)_ formulations of both oils with Smix of Tween 20^®^ and Transcutol HP^®^ at 2:1 ratio showed the highest DC, and their characteristics were within the selection criteria. The higher was the oil concentration, the more was the amount of AZM incorporated in the formulations. This finding is probably related to the higher solubility of AZM in the oil phase than to its solubility in the used surfactant (Figure 2). However, L-F1_(H)_ formulation (prepared with Capryol 90^®^ oil) showed higher drug content (60.42 ± 0.4 mg/mL) than L-F2_(H)_ formulation (prepared with Octanoic acid) (46.9 ± 0.674 mg/mL); this might be attributed to the higher solubility of AZM in Capryol 90^®^ than in octanoic acid oil.

AZM addition to the blank L-SEDDs formulations also caused some changes in their DS, *Đ*, ZP, and T% characteristics (Table 4 and Table 5). These changes might be linked to the entering of the drug molecule into the interfacial surface where surfactant molecules exist [98]. Unlike formulations constructed with octanoic acid, formulations with Capryol 90^®^ oil showed characteristics robustness upon drug incorporation. Such observations were in line with previously reported data using high oil concentrations of 25% Capmul 808G EP/NF [107], as well as 30% of oil mixture (Maisine^®^ 35-1 and Labrafac^®^ CC (1:1)) [108,109].

Increasing oil concentration was associated with significant increases in DS and *Đ*, as well as a decrease in T% in all formulations. These effects could be attributed to the presence of an insufficient amount of surfactant and co-surfactant in the mixture to reduce the DS, and *Đ* values.

The ZP measurement results showed that the charge of the prepared L-SEDDs formulations was negative when dispersed in DW and SIF. This may be linked to the presence of free fatty acids in the oil phase and/or surfactant [110]. However, upon the dilution of L-SEDDs formulations with 0.1 mM HCl, the ZP charge was shifted to be positive. This could be due to the neutralization of fatty acids and their negatively charged hydroxyl groups (OH^−^) by the available positively charged hydrogens (H^+^) in such acidic medium.

Furthermore, increasing oil(s) concentration was associated with a significant increase in the ZP values of the formulations, yet, relatively lower ZP values were obtained for formulations constructed with octanoic acid oil compared with those constructed with Capryol 90^®^ oil. This might be related to the presence of a carboxyl group in the structure of octanoic acid [104]. The blank L-F1_(H)_ formulation as well as its drug-loaded form AZM-L-F1_(H)_ were shown to have the highest ZP values of (−23.03 ± 1.1 mV)) and (−26.47 ± 0.65 mV) respectively.

Based on all the previously described characterizations, L-F1_(H)_ and its drug-loaded AZM-L-F1_(H)_ formulations were found to meet the selection criteria and were selected for further study. The formulation was composed of 22.22%, 51.85%, and 25.93% (*v*/*v*) of Capryol 90^®^, Tween 20^®^, and Transcutol HP^®^ respectively.

### 3.5. Preparation of Solid Self-Emulsifying Drug Delivery System (S-SEDDs)

The water-insoluble solidifying agents such as Aerosil 200^®^ and calcium carbonate as well as the water-soluble solidifying agents such as mannitol and lactose are the most common solidifiers used for the production of S-SEDDs formulations [111,112,113,114,115,116]. In this study, the adsorption capacity (expressed as a weight ratio of L- SEDDs (L-F1_(H)_): Solidifying agent) of these solidifying agents were investigated. It was observed that each solidifying agent had a different adsorption capacity to yield a non-sticky solid powder of S-SEDDs formulations, as illustrated in Table 6.

Aerosil200^®^ was found to have the highest adsorption capacity of 2:1. Furthermore, it produced blank S-SEDDs formulation with the lowest DS (156.67 ± 1.5 nm) and *Đ* (0.62 ± 0.004). Accordingly, Aerosil200^®^ was selected as the optimized solidifier for production of the solid SEDDs (S-F1_(H)_) formulation. The characterization results of the solidified blank and AZM-loaded S-F1_(H)_ formulations are shown in Table 7.

The results of the solidification process showed that S-F1_(H)_ formulation had bigger DS, higher *Đ* values, and significantly lower AZM DC (38.79 ± 0.52 mg/g) in comparison to the L-F1_(H)_ formulation which had smaller DS, lower *Đ* values and higher AZM DC (60.42 ± 0.4 mg/mL). Such effects are linked to the need of using large amounts of Aerosil 200^®^ (~535.6 mg) to produce solid S-SEDDs formulation. The same pattern of characteristic change was previously reported upon solidifying L-SEDDs formulation [117].

### 3.6. Transmission Electron Microscope (TEM)

**Figure 4:**
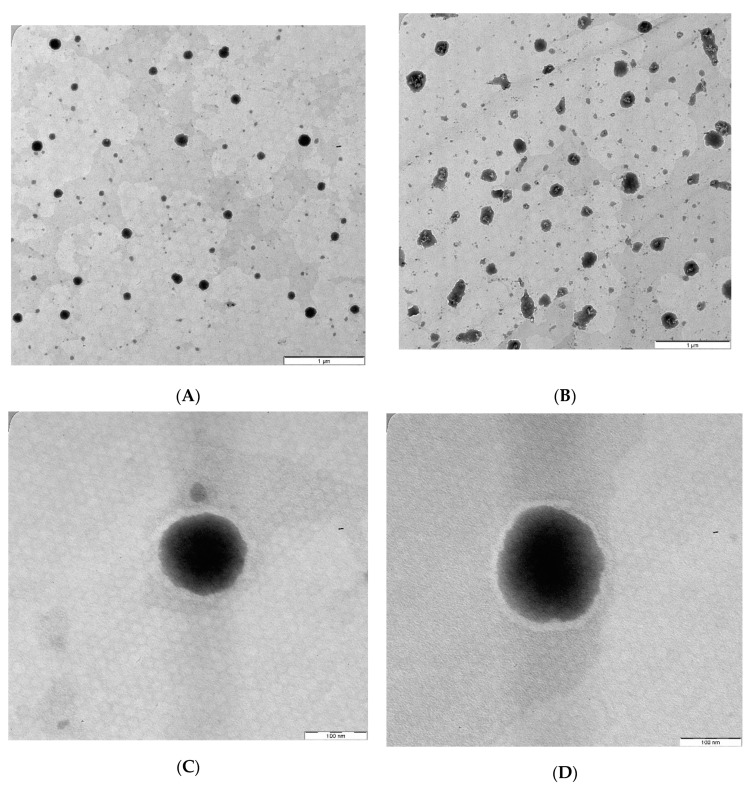
TEM images of the optimized AZM-loaded liquid and solid SEDDs formulations. (**A**) AZM-L-F1_(H)_ Dispersion; (**B**) AZM-S-F1_(H)_ Dispersion; (**C**) Individual droplet of AZM-L-F1_(H);_ (**D**) Individual droplet of AZM-S-F1_(H)._

The TEM images of the optimized AZM-loaded L-F1_(H)_ and S-F1_(H)_ formulations have revealed the formation of emulsion upon their dispersion (Figure 4A,B). The dispersed droplets of the prepared liquid and solid SEDDs formulations were spherical in shape with no signs of aggregation (Figure 4C,D). These findings are in line with previous TEM studies of liquid and solid SEDDS formulations [118,119,120,121].

### 3.7. Cell Culture

#### 3.7.1. Cytotoxicity Assay

Despite the fact that AZM is known to be cytocompatible [122,123] and the used excipients are considered relatively safe where they are used in the food, cosmetics or pharmaceutical industries (such as Transcutol^®^ HP [124], Tween 20^®^ [125], and Capryol 90^®^ [126]), However, the cytotoxicity study in this work was conducted before proceeding to the TEER assay to make sure that the SEDDs effects on TEER must not be linked to the cytotoxicity of the excipients or their combination in the developed SEDDs formulations. Caco-2 cell monolayer model was used in this study because it is a reliable in vitro model, an approved standard by USFDA and pharmaceutical companies to investigate the cytotoxicity and behavior of developed formulations and/or drug in the intestinal tract [127,128]. The MTT assay is one of the most popular in vitro cytotoxicity assays. Its mechanism is based on the conversion of the water-soluble thiazolyl blue tetrazolium bromide dye to an insoluble purple-colored formazan, which is quantified by a microplate photometer [129]. The amount of formed crystals and the obtained readings represent the metabolic activities, and thus the number of the present viable cells [129,130]. The selected concentrations of the finally optimized blank formulations were chosen so that both formulations would have the same drug concentration when later preforming the TEER evaluation.

**Figure 5:**
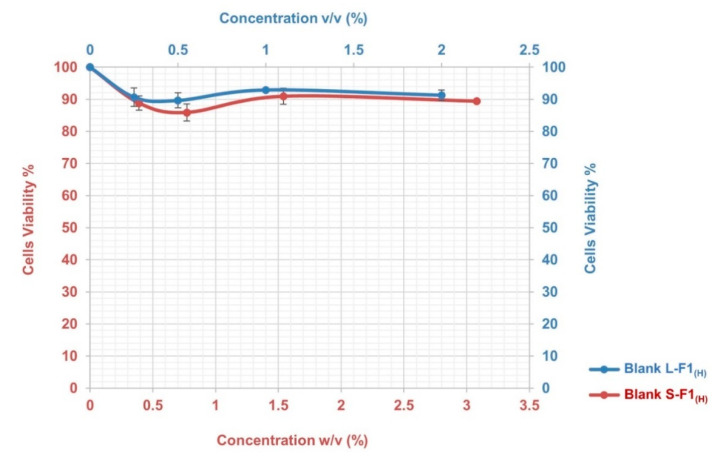
The cytotoxicity profile of Blank L-F1_(H)_, and Blank S-F1_(H)_ formulations at different concentrations. Mean ± SEM, n = 3.

The results of the MTT assay indicated that the blank L-F1_(H)_ and S-F1_(H)_ formulations had low cytotoxicity with no induced toxic effects during the 4 h incubation period and at the different studied concentrations. As shown in Figure 5, the cells viabilities were above ~90% and ~86% for the tested blank L-F1_(H)_ and S-F1_(H)_ formulations, respectively.

#### 3.7.2. Transepithelial Electrical Resistance Evaluation

**Figure 6:**
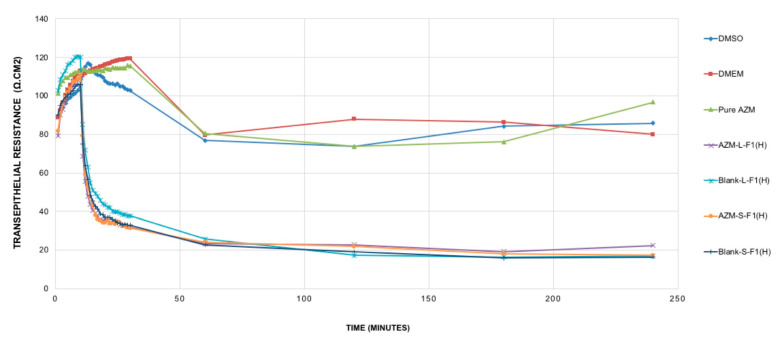
TEER resistance of the studied samples. Mean ± SEM, n = 3. DMSO: dimethyl sulfoxide, DMEM: Dulbecco’s modified eagle’s medium, AZM-L-F1_(H)_: Azithromycin-loaded liquid SEDDs, Blank L-F1_(H)_: Blank liquid SEDDS, Pure AZM: Pure Azithromycin, AZM-S-F1_(H)_: Azithromycin-loaded solid SEDDs, Blank S-F1_(H)_: Blank solid SEDDS.

The modulation of TJ is a potent strategy to improve hydrophobic drug delivery [131]; such modulations were related to the used formulations’ excipients, mostly the surfactants [132,133,134] and some fatty acids [42]. In general, the opening of the TJ is associated with a decrease of transepithelial electrical resistance (TEER) [135,136]. The TEER evaluation study results are shown in Figure 6.

It was revealed that pure AZM had no significant impact on TJ in the Caco-2 cell line model and within the studied period of 4 h. Such results are in contrast with previous studies that showed pure AZM could increase the TEER values in airway epithelial cells [21]. Such a difference in AZM behavior might be linked to the variations in cell lines’ type, source, passage number, and culturing conditions. These results are suggesting the need for more studies to understand the exact cellular mechanisms of AZM permeation and its relation to its bioavailability using different cell lines and in vivo levels. Upon treating the cells with the set samples, and during the first 10 min of the measurement period, all samples (AZM, controls, blank and drug-loaded formulations) showed almost no significant changes in the TEER values. After that, only blank and drug-loaded formulations of L-SEDDs and S-SEDDs were able to decrease the TEER values sharply. These declines were significant (*p* < 0.05) in comparison to the TEER values of pure AZM, and could be linked to the ability of the optimized formulations to modulate the intestinal tight junction, possibly due to the presence of Capryol 90^®^, which has a proven ability to produce an appreciable reversible opening of TJ [33], which in turn might offer better delivery of AZM via paracellular pathways [137]

### 3.8. The In Vitro Release Studies

**Figure 7:**
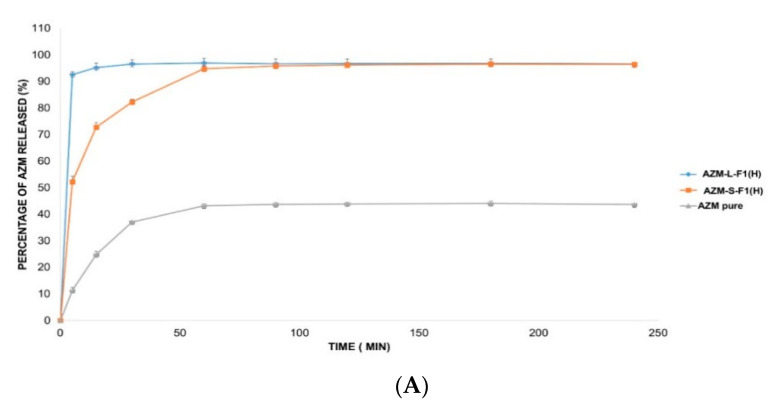

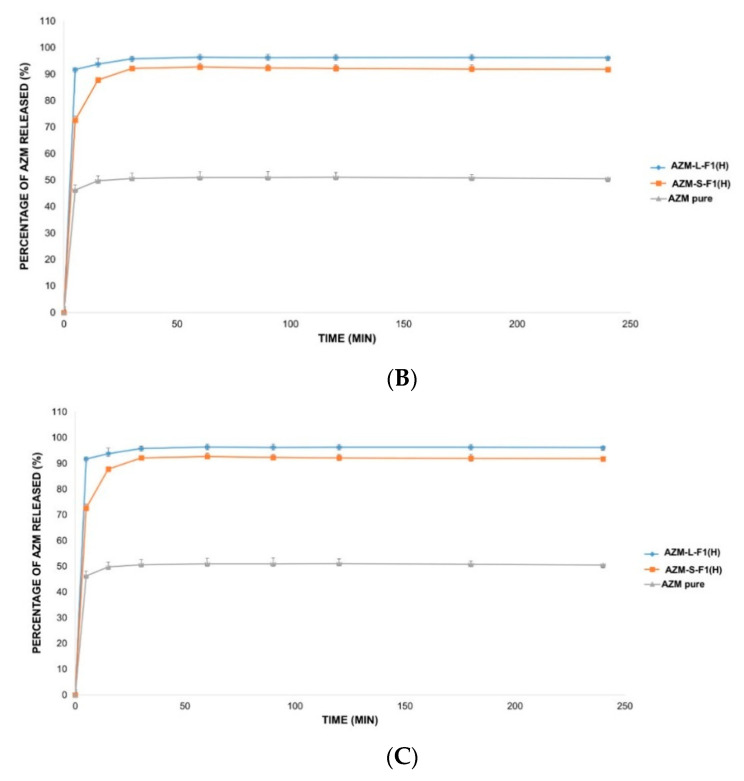
The in vitro release profile of AZM-L-F1_(H)_, AZM-S-F1_(H)_ formulations, and pure AZM in DW, HCl, and SIF, respectively. (**A**) The in vitro release profile in DW. (**B**) The in vitro release profile in 0.1 mM HCl (pH = 4). (**C**) The in vitro release profile in SIF pH = (6.8).

The in vitro release of AZM is usually conducted in a medium that mimics the intestinal pH such as phosphate buffer (pH = 6). This is because AZM is not sufficiently stable in acidic pH mediums such as simulated gastric fluids (SGF, pH = 1.2) [138]. Accordingly, three different release solutions were employed in this study including 0.1 mM HCl (pH = 4), DW (pH = 5.5), and SIF medium (pH = 6.8) [103,104,139]. Figure 7 represents the in vitro release profiles of AZM from AZM-L-F1_(H)_ and AZM-S-F1_(H)_ formulations, compared to the release of the pure AZM in the media of DW, 0.1 mM HCl, and SIF, respectively.

The liquid and solid SEDDs formulations were able to significantly (*p* < 0.05) increase the percentage of AZM release by 2.2-fold in DW, 1.9 and 1.8 folds in HCl, 1.7 and 1.6 folds in SIF, respectively in comparison with the pure AZM during the 4 h dissolution period.

The cumulative release percentages from the pure AZM were only 43.68 ± 0.84%, 50.57 ± 1.24% and 58.87 ± 2.23% in DW, HCl and SIF media respectively. In contrast, the AZM-L-F1_(H)_ formulation released > 90% of the AZM within the first 5 min of the dissolution in all the studied dissolution media including 96.65 ± 1.77%, 96.21 ± 1.06%, 98.49 ± 0.77% in DW, HCl, and SIF, respectively. While the AZM-S-F1_(H)_ formulation showed a slower release pattern whereby in 5 min, only 52.22%, 74.74%, and 70.01% were released in DW, HCL, and SIF, respectively. Unlike the L-SEDDs, S-SEDDs formulation needed 60 min, 30 min, and 15 min to reach > 90% of AZM in DW, HCL, and SIF. Such delay of AZM release from S-SEDDs formulation was in line with previous studies [34,140]. The researchers linked it to that the S-SEDDs formulation needed more step such as desorption of the adsorbed SEDDs from the Aerosol 200^®^ during dissolution process [141]. Based on these results, the developed formulations were able to significantly enhance the dissolution rate of AZM in comparison to the pure drug powder.

### 3.9. Stability Studies

#### 3.9.1. Physical Stability

The physical appearance of AZM-L-F1_(H)_ and AZM-S-F1_(H)_ formulations were maintained unchanged during the 3 months of storage under the different conditions at the set different storage conditions.

#### 3.9.2. Droplet Size, *Đ*, and Zeta Potential

The results of the stability studies of AZM-L-F1_(H)_ and AZM-S-F1_(H)_ formulations under the three different storage conditions are shown in Table 8 and Table 9. Both formulations showed better stability in the refrigerator condition (4 ± 2 °C) when compared with the other studied storage conditions (i.e., room and humidity chamber conditions). For instance, after three months of storage in the refrigerator, the ZP values of both formulations were maintained unchanged, while there were slight but significant increases in DS of ~8 and 5 nm, respectively. The same increment patterns were seen in the *Đ* values of the AZM-L-F1_(H)_ formulation at the three storage conditions. In contrast, there were no changes in the *Đ* values of the AZM-S-F1_(H)_ formula under all the storage conditions during the three months period.

#### 3.9.3. Chemical Stability

All formulations showed no statistically significant changes in DC% when stored in the refrigerator (*p* < 0.05). However, under the room storage condition (25 ± 2 °C/60 ± 5% RH), only AZM-S-F1_(H)_ formulation was stable with no significant decreases in the DC% for the period of three months. On the other hand, decreases in DC% were observed in both formulations when stored at the humidity chamber (40 ± 2 °C/75 ± 5% RH).

## 4. Conclusions

Liquid and solid emulsifying SEDDS were successfully developed and optimized. The medium carbon chain triglycerides (MCT) were better than the long carbon chain triglycerides (LCT) as solubilizing agents for AZM. The hydrophobic solidifying agent (Aerosil 200^®^) was found to have the best solidification capacity among the studied solidifying agents to produce solid SEDDs. In this study, the main suggested reasons behind the low oral bioavailability of AZM were addressed, whereby both the liquid and solid SEDDs formulations improved the solubility and dissolution rate of the drug. However, more studies are required to specify the exact reasons that are responsible for the low oral bioavailability of this drug. The cytotoxicity study using MTT assay revealed that the AZM-SEDDs formulations had a low toxicity profile. Furthermore, the transepithelial electrical resistance (TEER) evaluations showed that both formulations had the abilities to open the tight epithelial junction. The formulations were relatively stable at the refrigerator storage condition for 3 months. Therefore, it is suggested that the developed liquid and solid self-emulsifying drug delivery systems are potential nanocarriers for the oral delivery of AZM.

## Figures and Tables

**Table 1:** Screened excipients’ role, characteristics, and previously reported cellular activities.

Name	Role	HLB	Chain Length	Reported Cellular Activity (s)	References
Capryol 90^®^	Oil	5	Medium-chain (C8)	TJ opening	[33,40,41]
Castor Oil		-	Long-chain (C18)	-	-
Octanoic acid (caprylic acid or caprylate)		-	Medium-chain (C8)	TJ opening	[42,43]
Palm Oil		-	Long-chain. Mixture of C16 and C18	-	-
Pureco 76^®^		-	Medium-chain (C8–C10)	-	-
Cremophor EL^®^	Surfactant	12–14	Medium-chain C8	TJ opening and P-gp inhibitor	[44]
Cremophor RH40^®^		14–16	Medium-chain C10	TJ opening and P-gp inhibitor	[45]
Labrafac PG^®^		1	Medium-chain. Mixture of C8 and C10	-	-
Labrafil^®^ M 1944 CS		9	Long-chain C18	-	-
Labrasol^®^		12–14	Medium-chain (C8–C10)	TJ opening and P-gp inhibitor	[44,46]
Tween 20^®^		16.7	Medium-chain C12	P-gp inhibitor	[47,48,49]
Tween 80^®^		15	Long-chain C18	TJ opening and P-gp inhibitor	[50,51,52]
Span 20^®^	Co-surfactant	8.6	Medium-chain C12	P-gp inhibitor	[53]
Span 80^®^		4.3	Long-chain C18	-	-
Transcutol HP^®^		-	-	P-gp inhibitor	[54]

(HLB, Hydrophile-lipophile balance; TJ, Tight Junctions; P-gp, P-glycoprotein).

**Table 2:** The combination of the selected components in liquid self-emulsifying drug delivery systems (L-SEDDs) formulations.

No.	Formulation Code	Oil	Surfactant	Co-Surfactant
1	A1	Capryol 90^®^	Labrasol^®^	Transcutol HP^®^
2	B1		Labrasol^®^	Span 20^®^
3	C1		Tween 20^®^	Transcutol HP^®^
4	D1		Tween 20^®^	Span 20^®^
5	A2	Octanoic acid	Labrasol^®^	Transcutol HP^®^
6	B2		Labrasol^®^	Span 20^®^
7	C2		Tween 20^®^	Transcutol HP^®^
8	D2		Tween 20^®^	Span 20^®^

**Table 3:** The selected L-SEDDs components’ concentrations and droplet size (DS) out of the constructed pseudo-ternary diagrams. Mean ± SD, n = 3.

Oil %	Surfactant %	Co-Surfactant %	DS (nm)
**Diagram 1: Capryol 90^®^, Tween 20^®^, and Transcutol HP^®^ at Smix ratio of 2:1**			
11.11111	59.25926	29.62963	113.6 ± 1.3
16.66667	55.55556	27.77778	126.03 ± 1.3
22.22222	51.85185	25.92593	139.2 ± 0.69
**Diagram 2: Capryol 90^®^, Tween 20^®^, and Transcutol HP^®^ at a Smix ratio of 5:1**			
11.11111	74.07407	14.81481	56.17 ± 0.76
16.66667	69.44444	13.88889	76 ± 1
22.22222	64.81481	12.96296	94 ± 1
**Diagram 3: Capryol 90^®^, Tween 20^®^, and Transcutol HP^®^ at a Smix ratio of 6:1**			
11.11111	76.19048	12.69841	10.1 ± 0.3
16.66667	71.42857	11.90476	12.13 ± 0.31
22.22222	66.66667	11.11111	60.27 ± 1.1
**Diagram 4: Octanoic acid, Tween 20^®^, and Transcutol HP^®^ at a Smix ratio of 2:1**			
11.11111	59.25926	29.62963	125.1 ± 1.1
16.66667	55.55556	27.77778	140.03 ± 0.95
22.22222	51.85185	25.92593	145 ± 1
**Diagram 5: Octanoic acid, Tween 20^®^, and Transcutol HP^®^ at a Smix ratio of 3:1**			
11.11111	66.66667	22.22222	98.13 ± 0.91
16.66667	62.5	20.83333	112.73 ± 1.14
22.22222	58.33333	19.44444	144.23 ± 0.93
**Diagram 6: Octanoic acid, Tween 20^®^, and Transcutol HP^®^ at a Smix ratio of 4:1**			
11.11111	71.11111	17.77778	86.37 ± 1.1
16.66667	66.66667	16.66667	103.6 ± 0.36
22.22222	62.22222	15.55556	112.2 ± 0.72
**Diagram 7: Octanoic acid, Tween 20^®^, and Transcutol HP^®^ at a Smix ratio of 5:1.**			
11.11111	74.07407	14.81481	63.67 ± 1.5
16.66667	69.44444	13.88889	87.1 ± 1.2
22.22222	64.81481	12.96296	109.07 ± 0.86
**Diagram 8: Octanoic acid, Tween 20^®^, and Transcutol HP^®^ at a Smix ratio of 6:1**			
11.11111	76.19048	12.69841	10.13 ± 0.15
16.66667	71.42857	11.90476	13.43 ± 0.35
22.22222	66.66667	11.11111	97.43 ± 1.09

(DS, Droplet size; Smix, Surfactant to co-surfactant ratio).

**Table 4:** Characterization of blank and AZM-L-F1 formulations at Capryol 90^®^ oil concentrations of low, medium, and high analyzed in the three diluents. Mean ± SD, n = 3.

Blank L-SEDDs					AZM-L-SEDDs			
**DW**								
Code	DS (nm)	*Đ*	ZP (mV)	T%	DS (nm)	*Đ*	ZP (mV)	T%
L-F1_(L)_	112.63 ± 1.02	0.56 ± 0.05	−18.5 ± 0.96	90.17 ± 0.56	112.17 ± 1.26	0.41 ± 0.01	−21.73 ± 0.96	91.51 ± 0.19
L-F1_(M)_	125.4 ± 1.02	0.57 ± 0.002	−23.3 ± 1.4	90.01 ± 0.66	124.9 ± 1.65	0.47 ± 0.01	−23.1 ± 0.96	90.33 ± 0.31
L-F1_(H)_	138.6 ± 0.85	0.59 ± 0.009	−23.03 ± 1.1	90.1 ± 0.11	141.57 ± 1.1	0.52 ± 0.004	−26.47 ± 0.65	90.1 ± 0.1
**0.1 mM HCl (pH = 4)**								
L-F1_(L)_	116.83 ± 1.3	0.56 ± 0.004	1.24 ± 1.5	94.75 ± 0.11	97.6 ± 1.6	0.52 ± 0.003	0.33 ± 1.7	98.2 ± 0.23
L-F1_(M)_	112.63 ± 1.4	0.54 ± 0.01	−0.89 ± 0.28	94.45 ± 0.11	91.23 ± 0.76	0.54 ± 0.003	−0.896 ± 0.35	98.04 ± 0.06
L-F1_(H)_	93.5 ± 1.3	0.62 ± 0.002	0.24 ± 0.47	94.75 ± 0.05	86.6 ± 1.44	0.52 ± 0.003	0.567 ± 2.2	97.99 ± 0.09
**SIF (pH = 6.8)**								
L-F1_(L)_	89.43 ± 0.53	0.628 ± 0.005	−3.23 ± 0.11	93.3 ± 0.1	166.27 ± 1.96	0.824 ± 0.01	−3.27 ± 0.55	97.47 ± 0.08
L-F1_(M)_	86.63 ± 1.25	0.63 ± 0.01	−5.86 ± 0.68	93.48 ± 0.13	154.67 ± 2.1	0.83 ± 0.02	−6.05 ± 1.3	95.23 ± 0.12
L-F1_(H)_	95.47 ± 1.7	0.657 ± 0.04	−5.28 ± 0.89	93.38 ± 0.13	148.1 ± 1.6	0.68 ± 0.006	−5.97 ± 1.8	94.55 ± 0.48

(Blank L-SEDDs, Blank liquid self-emulsifying drug delivery systems; AZM-L-SEDDs, Azithromycin-loaded self-emulsifying drug delivery systems; DW, Distilled water; DS, Droplet size; *Đ*, Dispersity; ZP, Zeta potential; T%, Transmittance percentage; HCl, Hydrochloric acid).

**Table 5:** Characterization of blank and AZM-L-F2 formulations at Octanoic acid oil concentrations of low, medium, and high analyzed in the three diluents. Mean ± SD, n = 3.

Blank L-SEDDs					AZM-L-SEDDs			
**DW**								
Code	DS (nm)	*Đ*	ZP (mV)	T%	DS (nm)	*Đ*	ZP (mV)	T%
L-F2_(L)_	124.93 ± 1.5	0.64 ± 0.003	−17 ± 0.76	89.44 ± 0.26	143.1 ± 1.4	0.84 ± 0.02	−16.3 ± 0.87	90.47 ± 0.24
L-F2_(M)_	139.73 ± 1.31	0.59 ± 0.005	−17.1 ± 0.53	88.69 ± 0.08	148.43 ± 1.3	0.71 ± 0.003	−17.77 ± 0.96	89.03 ± 0.15
L-F2_(H)_	143.9 ± 1.64	0.68 ± 0.002	−18.23 ± 0.59	88.83 ± 0.32	267.8 ± 1.9	1 ± 0	−18.43 ± 0.97	77.96 ± 0.48
**0.1 mM HCl (pH = 4)**								
L-F2_(L)_	104.65 ± 0.5	0.45 ± 0.002	0.27 ± 1.8	94.75 ± 0.1	113.7 ± 1.8	0.61 ± 0.07	0.086 ± 1.1	97.17 ± 0.55
L-F2_(M)_	113.53 ± 1.8	0.47 ± 0.003	−2.6 ± 0.4	93.77 ± 0.04	127.6 ± 1.5	0.54 ± 0.009	−0.58 ± 0.83	96.56 ± 0.57
L-F2_(H)_	130.37 ± 0.6	0.55 ± 0.002	0.24 ± 0.5	90.75 ± 0.07	136.7 ± 1.9	0.54 ± 0.02	0.62 ± 0.8	91.76 ± 0.58
**SIF (pH = 6.8)**								
L-F2_(L)_	126.77 ± 1.2	1 ± 0	−3.61 ± 0.5	93.03 ± 0.11	149.77 ± 1.5	0.68 ± 0.01	−1.53 ± 0.17	89.1 ± 0.68
L-F2_(M)_	115.6 ± 1.1	1 ± 0	−4.18 ± 0.7	93.47 ± 0.12	159.47 ± 0.93	1 ± 0	−4.16 ± 1.02	86.62 ± 0.29
L-F2_(H)_	127.2 ± 1.4	1 ± 0	−4.44 ± 0.7	93.35 ± 0.13	226.3 ± 2.01	0.99 ± 0.006	−3.88 ± 0.69	75.02 ± 0.08

(Blank L-SEDDs, Blank liquid self-emulsifying drug delivery systems; AZM-L-SEDDs, Azithromycin-loaded self-emulsifying drug delivery systems DW, Distilled water; DS, Droplet size; *Đ*, Dispersity; ZP, Zeta potential; T%, Transmittance percentage; HCl, Hydrochloric acid).

**Table 6:** Adsorption capacity and characterization of blank S-SEDDs (S-F1_(H)_) formulation. Mean ± SD, n = 3.

Solidifying Agent	Adsorption Capacity (L-F1_(H)_): Solidifying Agent	DS (nm)	*Đ*	ZP (mV)
Calcium carbonate	(1:4)	1863.67 ± 10.3	1 ± 0	−14.47 ± 1.001
Aerosil 200^®^	(2:1)	156.67 ± 1.5	0.62 ± 0.004	−21.7 ± 1.4
Lactose	(1:3)	384.7 ± 4.04	1 ± 0	−40.33 ± 1.07
Mannitol	(1:2)	845.63 ± 6.2	1 ± 0	−23.4 ± 1.3

(DS, Droplet size; *Đ*, Dispersity; ZP, Zeta potential).

**Table 7:** Characterization of the optimized blank and AZM loaded S-F1(H) formulations in DW, 0.1 mM HCl, and SIF diluents. Mean ± SD, n = 3.

Blank S-SEDDs (S-F1_(H)_)			AZM S-SEDDs (S-F1_(H)_)		
**DW**					
DS (nm)	*Đ*	ZP (mV)	DS (nm)	*Đ*	ZP (mV)
157.1 ± 1.85	0.61 ± 0.01	−22.67 ± 2.3	155.3 ± 1.91	0.62 ± 0.03	−19.43 ± 0.15
**0.1 mM HCl (pH = 4)**					
139.03 ± 1.8	1 ± 0	2.1 ± 0.2	136.6 ± 1.9	0.73 ± 0.01	0.88 ± 0.02
**SIF (pH = 6.8)**					
195.32 ± 1.9	1 ± 0	−5.93 ± 1.1	191.5 ± 1.4	1 ± 0	−7.43 ± 0.81

(DW, Distilled water; DS, Droplet size; *Đ*, Dispersity; ZP, Zeta potential; HCl, Hydrochloric acid; SIF, Simulated intestinal fluid).

**Table 8:** Stability studies for AZM-L-F1_(H)_ formulation under the three different storage conditions. Mean ± SD, n = 3.

Parameters	Time (Months)				
	0	0.5	1	2	3
**Refrigerator (4 ± 2 °C)**					
**Physical stability**					
DS (nm)	141.23 ± 0.38	141.6 ± 0.99	142 ± 1.15	146.87 ± 1.3	149.97 ± 1.16
*Đ*	0.528 ± 0.006	0.522 ± 0.01	0.534 ± 0.005	0.541 ± 0.009	0.56 ± 0.01
ZP (mV)	−26.78 ± 1.056	−27.43 ± 1.23	−26.73 ± 1.07	−25.69 ± 0.56	−26.3 ± 0.5
**Chemical stability**					
DC (%)	99.843 ± 0.353	99.79 ± 0.45	99.66 ± 0.162	99.37 ± 0.12	99.43 ± 0.22
**Room condition (25 ± 2 °C/60 ± 5% RH)**					
**Physical stability**					
DS (nm)	141.23 ± 0.38	142.03 ± 0.42	143.7 ± 1.31	148.23 ± 1.23	156.83 ± 1.5
*Đ*	0.528 ± 0.006	0.524 ± 0.04	0.53 ± 0.002	0.54 ± 0.003	0.552 ± 0.01
ZP (mV)	−26.78 ± 1.056	−26.7 ± 1.1	−26.37 ± 0.42	−25.73 ± 51	−25.47 ± 0.65
**Chemical stability**					
DC (%)	99.843 ± 0.353	99.65 ± 0.2	99.42 ± 0.14	99.241 ± 0.17	99.11 ± 0.12
**Humidity chamber (40 ± 2 °C/75 ± 5% RH)**					
**Physical stability**					
DS (nm)	141.23 ± 0.38	142.03 ± 1.16	147.55 ± 1.05	149.57 ± 1.37	166.63 ± 1.56
*Đ*	0.528 ± 0.006	0.529 ± 0.007	0.53 ± 0.003	0.542 ± 0.007	0.613 ± 0.05
ZP (mV)	−26.78 ± 1.056	−26.93 ± 0.15	−25.97 ± 0.7	−25.67 ± 0.32	−23.3 ± 0.56
**Chemical stability**					
DC (%)	99.843 ± 0.353	99.74 ± 0.13	99.12 ± 0.08	98.95 ± 0.1	98.58 ± 0.43

(DS, Droplet size; *Đ*, Dispersity; ZP, Zeta potential; DC%, Drug content percentage; RH, Relative humidity).

**Table 9:** Stability studies for AZM-S-F1_(H)_ formulation under the three different storage conditions. Mean ± SD, n = 3.

Parameters	Time (Months)				
	0	0.5	1	2	3
**Refrigerator (4 ± 3 °C)**					
**Physical stability**					
DS (nm)	155.93 ± 1.39	155.13 ± 1.35	156.27 ± 1.19	157.33 ± 0.59	159.2 ± 0.78
*Đ*	0.64 ± 0.04	0.65 ± 0.015	0.64 ± 0.015	0.66 ± 0.021	0.66 ± 0.01
ZP (mV)	−19.28 ± 0.45	−19.23 ± 0.32	−19.27 ± 0.45	−18.97 ± 0.75	−18.49 ± 0.6
**Chemical stability**					
DC (%)	98.599 ± 0.32	98.52 ± 0.38	98.41 ± 0.57	98.37 ± 0.2	98.09 ± 0.5
**Room condition (25 ± 2 °C/60 ± 5% RH)**					
**Physical stability**					
DS (nm)	155.93 ± 1.39	155.8 ± 1.67	155.6 ± 1.25	158.3 ± 1.25	161.55 ± 1.1
*Đ*	0.64 ± 0.04	0.627 ± 0.025	0.65 ± 0.02	0.647 ± 0.03	0.68 ± 0.006
ZP (mV)	−19.28 ± 0.45	−19.15 ± 0.53	−18.97 ± 0.31	−18.77 ± 0.31	−16.27 ± 0.45
**Chemical stability**					
DC (%)	98.599 ± 0.32	98.42 ± 0.12	98.34 ± 0.36	98.06 ± 0.33	97.92 ± 0.43
**Humidity chamber (40 ± 2 °C/75 ± 5% RH)**					
**Physical stability**					
DS (nm)	155.93 ± 1.39	156.4 ± 0.6	158 ± 1	162.37 ± 1.1	164.1 ± 1.02
*Đ*	0.64 ± 0.04	0.65 ± 0.01	0.653 ± 0.015	0.657 ± 0.015	0.603 ± 0.006
ZP (mV)	−19.28 ± 0.45	−19.03 ± 0.4	−18.9 ± 0.3	−18.63 ± 0.42	−15.3 ± 0.56
**Chemical stability**					
DC (%)	98.599 ± 0.32	98.44 ± 0.28	98.014 ± 0.4	97.86 ± 0.37	97.48 ± 0.58

(DS, Droplet size; *Đ*, Dispersity; ZP, Zeta potential; DC%, Drug content percentage; RH, Relative humidity).

## References

[1] Al-Achi A., Gupta M.R., Stagner W.C. (2013). Integrated Pharmaceutics: Applied Preformulation, Product Design, and Regulatory Science.

[2] Torne S.R., Sheela A., Sarada N.C. (2018). A Review on Oral Liquid as an Emerging Technology in Controlled Drug Delivery System. Curr. Pharm. Des..

[3] Ghasemiyeh P., Mohammadi-Samani S. (2018). Solid lipid nanoparticles and nanostructured lipid carriers as novel drug delivery systems: Applications, advantages and disadvantages. Res. Pharm. Sci..

[4] Laksitorini M., Prasasty V.D., Kiptoo P.K., Siahaan T.J. (2014). Pathways and progress in improving drug delivery through the intestinal mucosa and blood-brain barriers. Ther. Deliv..

[5] (2015). The United States Pharmacopoeia and National Formulary.

[6] Imperi F., Leoni L., Visca P. (2014). Antivirulence activity of azithromycin in Pseudomonas aeruginosa. Front. Microbiol..

[7] World Health Organization (2013). WHO Model List of Essential Medicines. https://www.who.int/medicines/publications/essentialmedicines/en/.

[8] Peters D.H., Friedel H.A., McTavish D. (1992). Azithromycin. A review of its antimicrobial activity, pharmacokinetic properties and clinical efficacy. Drugs.

[9] Williams J.D. (1991). Spectrum of Activity of Azithromycin. Eur. J. Clin. Microbiol. Infect. Dis..

[10] Gautret P., Lagier J.-C., Parola P., Hoang V.T., Meddeb L., Mailhe M., Doudier B., Courjon J., Giordanengo V., Vieira V.E. (2020). Hydroxychloroquine and azithromycin as a treatment of COVID-19: Results of an open-label non-randomized clinical trial. Int. J. Antimicrob. Agents.

[11] Adeli E., Mortazavi S.A. (2014). Design, formulation and evaluation of Azithromycin binary solid dispersions using Kolliphor series for the solubility and in vitro dissolution rate enhancement. Int. J. Pharm. Investig..

[12] Aucamp M., Odendaal R., Liebenberg W., Hamman J. (2015). Amorphous azithromycin with improved aqueous solubility and intestinal membrane permeability. Drug Dev. Ind. Pharm..

[13] Adrjanowicz K., Zakowiecki D., Kaminski K., Hawelek L., Grzybowska K., Tarnacka M., Paluch M., Cal K. (2012). Molecular dynamics in supercooled liquid and glassy states of antibiotics: Azithromycin, clarithromycin and roxithromycin studied by dielectric spectroscopy. Advantages given by the amorphous state. Mol. Pharm..

[14] Montejo-Bernardo J., García-Granda S., Bayod M., Llorente I., Llavona L. (2005). An easy and general method for quantifying Azithromycin dihydrate in a matrix of amorphous Azithromycin. Arkivoc.

[15] Arora S.C., Sharma P.K., Irchhaiya R., Khatkar A., Singh N., Gagoria J. (2010). Development, characterization and solubility study of solid dispersions of azithromycin dihydrate by solvent evaporation method. J. Adv. Pharm. Technol..

[16] Tung N.-T., Tran C.-S., Nguyen T.-L., Hoang T., Trinh T.-D., Nguyen T.-N. (2018). Formulation and biopharmaceutical evaluation of bitter taste masking microparticles containing azithromycin loaded in dispersible tablets. Eur. J. Pharm. Biopharm..

[17] Luke D.R., Foulds G. (1997). Disposition of oral azithromycin in humans. Clin. Pharmacol. Ther..

[18] Garver E., Hugger E.D., Shearn S.P., Rao A., Dawson P.A., Davis C.B., Han C. (2008). Involvement of intestinal uptake transporters in the absorption of azithromycin and clarithromycin in the rat. Drug Metab. Dispos..

[19] Nožinić D., Milić A., Mikac L., Ralić J., Padovan J., Antolović R. (2010). Assessment of Macrolide Transport Using Pampa, Caco-2 and Mdckii-Hmdr1 assays. Croat. Chem. Acta.

[20] Fohner A.E., Sparreboom A., Altman R.B., Klein T.E. (2017). PharmGKB summary: Macrolide antibiotic pathway, pharmacokinetics/pharmacodynamics. Pharm. Genom..

[21] Asgrimsson V., Gudjonsson T., Gudmundsson G.H., Baldursson O. (2006). Novel Effects of Azithromycin on Tight Junction Proteins in Human Airway Epithelia. Antimicrob. Agents Chemother..

[22] Arason A.J., Joelsson J.P., Valdimarsdottir B., Sigurdsson S., Gudjonsson A., Halldorsson S., Johannsson F., Rolfsson O., Lehmann F., Ingthorsson S. (2019). Azithromycin induces epidermal differentiation and multivesicular bodies in airway epithelia. Respir. Res..

[23] Salama N.N., Eddington N.D., Fasano A. (2006). Tight junction modulation and its relationship to drug delivery. Adv. Drug Deliv. Rev..

[24] U.S. National Center for Biotechnology Information Database (2016). Azithromycin. https://pubchem.ncbi.nlm.nih.gov/compound/Azithromycin.

[25] Adeli E. (2016). Preparation and evaluation of azithromycin binary solid dispersions using various polyethylene glycols for the improvement of the drug solubility and dissolution rate. Braz. J. Pharm..

[26] Hou C.-D., Wang J.-X., Le Y., Zou H.-K., Zhao H. (2012). Preparation of azithromycin nanosuspensions by reactive precipitation method. Drug Dev. Ind. Pharm..

[27] Zhong M., Feng Y., Liao H., Hu X., Wan S., Zhu B., Zhang M., Xiong H., Zhou Y., Zhang J. (2014). Azithromycin Cationic Non-Lecithoid Nano/Microparticles Improve Bioavailability and Targeting Efficiency. Pharm. Res..

[28] Pouton C.W. (2000). Lipid formulations for oral administration of drugs: Non-emulsifying, self-emulsifying and ‘self-microemulsifying’ drug delivery systems. Eur. J. Pharm. Sci..

[29] Pouton C.W. (2006). Formulation of poorly water-soluble drugs for oral administration: Physicochemical and physiological issues and the lipid formulation classification system. Eur. J. Pharm. Sci..

[30] Yakushiji K., Sato H., Ogino M., Suzuki H., Seto Y., Onoue S. (2020). Self-Emulsifying Drug Delivery System of Celecoxib for Avoiding Delayed Oral Absorption in Rats with Impaired Gastric Motility. AAPS PharmSciTech.

[31] Potharaju S., Mutyam S.K., Liu M., Green C., Frueh L., Nilsen A., Pou S., Winter R., Riscoe M.K., Shankar G. (2020). Improving solubility and oral bioavailability of a novel antimalarial prodrug: Comparing spray-dried dispersions with self-emulsifying drug delivery systems. Pharm. Dev. Technol..

[32] Mazzeti A.L., Oliveira L.T., Gonçalves K.R., Schaun G.C., Mosqueira V.C.F., Bahia M.T. (2020). Benznidazole self-emulsifying delivery system: A novel alternative dosage form for Chagas disease treatment. Eur. J. Pharm. Sci..

[33] Ukai H., Iwasa K., Deguchi T., Morishita M., Katsumi H., Yamamoto A. (2020). Enhanced Intestinal Absorption of Insulin by Capryol 90, a Novel Absorption Enhancer in Rats: Implications in Oral Insulin Delivery. Pharmaceutics.

[34] Kamal M.M., Salawi A., Lam M., Nokhodchi A., Abu-Fayyad A., El Sayed K.A., Nazzal S. (2020). Development and characterization of curcumin-loaded solid self-emulsifying drug delivery system (SEDDS) by spray drying using Soluplus^®^ as solid carrier. Powder Technol..

[35] Tengshe S.D., Karande K.M. (2020). A Review on Self Micro-Emulsifying Drug Delivery System: A Tool for Solubility Enhancement. Int. J. Res. Anal. Rev..

[36] Aloisio C., Bueno M.S., Ponte M.P., Paredes A., Palma S.D., Longhi M. (2019). Development of solid self-emulsifying drug delivery systems (SEDDS) to improve the solubility of resveratrol. Ther. Deliv..

[37] Assi R.A., Darwis Y., Abdulbaqi I.M., Asif S.M. (2017). Development and validation of a stability-indicating RP-HPLC method for the detection and quantification of azithromycin in bulk, and selfemulsifying drug delivery system (SEDDs) formulation. J. Appl. Pharm..

[38] Gurram A.K., Deshpande P.B., Kar S.S., Nayak U.Y., Udupa N., Reddy M.S. (2015). Role of Components in the Formation of Self-microemulsifying Drug Delivery Systems. Indian J. Pharm. Sci..

[39] Ye J., Wu H., Huang C., Lin W., Zhang C., Huang B., Lu B., Xu H., Li X., Long X. (2019). Comparisons of in vitro Fick’s first law, lipolysis, and in vivo rat models for oral absorption on BCS II drugs in SNEDDS. Int. J. Nanomed..

[40] Sobhani H., Tarighi P., Ostad S.N., Shafaati A., Nafissi-Varcheh N., Aboofazeli R. (2018). Rapamycin-Loaded, Capryol(TM) 90 and Oleic Acid Mediated Nanoemulsions: Formulation Development, Characterization and Toxicity Assessment. Iran. J. Pharm. Res..

[41] Nasr A.M., Qushawy M.K., Elkhoudary M.M., Gawish A.Y., Elhady S.S., Swidan S.A. (2020). Quality by Design for the Development and Analysis of Enhanced In-Situ Forming Vesicles for the Improvement of the Bioavailability of Fexofenadine HCl in Vitro and in Vivo. Pharmaceutics.

[42] Lindmark T., Kimura Y., Artursson P. (1998). Absorption enhancement through intracellular regulation of tight junction permeability by medium chain fatty acids in Caco-2 cells. J. Pharmacol. Exp. Ther..

[43] Suzuki T. (2020). Regulation of the intestinal barrier by nutrients: The role of tight junctions. Anim. Sci. J..

[44] Veeravalli V., Cheruvu H.S., Srivastava P., Vamsi Madgula L.M. (2020). Three-dimensional aspects of formulation excipients in drug discovery: A critical assessment on orphan excipients, matrix effects and drug interactions. J. Pharm. Anal..

[45] Zhao G., Huang J., Xue K., Si L., Li G. (2013). Enhanced intestinal absorption of etoposide by self-microemulsifying drug delivery systems: Roles of P-glycoprotein and cytochrome P450 3A inhibition. Eur. J. Pharm. Sci..

[46] Shen Y., Lu Y., Jv M., Hu J., Li Q., Tu J. (2011). Enhancing effect of Labrasol on the intestinal absorption of ganciclovir in rats. Drug Dev. Ind. Pharm..

[47] Gurjar R., Chan C.Y.S., Curley P., Sharp J., Chiong J., Rannard S., Siccardi M., Owen A. (2018). Inhibitory Effects of Commonly Used Excipients on P-Glycoprotein in Vitro. Mol. Pharm..

[48] Yang S., Liu J., Chen Y., Jiang J. (2012). Reversal effect of Tween-20 on multidrug resistance in tumor cells in vitro. Biomed. Pharmacother..

[49] Ma R., Li G., Wang X., Bi Y., Zhang Y. (2020). Inhibitory effect of sixteen pharmaceutical excipients on six major organic cation and anion uptake transporters. Xenobiotica.

[50] Lin H., Gebhardt M., Bian S., Kwon K.A., Shim C.K., Chung S.J., Kim D.D. (2007). Enhancing Effect of Surfactants on Fexofenadine Hcl Transport across the Human Nasal Epithelial Cell Monolayer. Int. J. Pharm..

[51] Zhang H., Yao M., Morrison R.A., Chong S. (2003). Commonly used surfactant, Tween 80, improves absorption of P-glycoprotein substrate, digoxin, in rats. Arch. Pharm. Res..

[52] Prabhakar K., Afzal S.M., Surender G., Kishan V. (2013). Tween 80 containing lipid nanoemulsions for delivery of indinavir to brain. Acta Pharm. Sin. B.

[53] Yamagata T., Kusuhara H., Morishita M., Takayama K., Benameur H., Sugiyama Y. (2007). Effect of excipients on breast cancer resistance protein substrate uptake activity. J. Control. Release.

[54] Hong J.Y., Kim J.K., Song Y.K., Park J.S., Kim C.K. (2006). A new self-emulsifying formulation of itraconazole with improved dissolution and oral absorption. J. Control. Release.

[55] Khames A. (2019). Formulation and Characterization of Eplerenone Nanoemulsion Liquisolids, An Oral Delivery System with Higher Release Rate and Improved Bioavailability. Pharmaceutics.

[56] Liu J., Hirschberg C., Fanø M., Mu H., Müllertz A. (2020). Evaluation of self-emulsifying drug delivery systems for oral insulin delivery using an in vitro model simulating the intestinal proteolysis. Eur. J. Pharm. Sci..

[57] Pal S., Mittapelly N., Husain A., Kushwaha S., Chattopadhyay S., Kumar P., Ramakrishna E., Kumar S., Maurya R., Sanyal S. (2020). A butanolic fraction from the standardized stem extract of Cassia occidentalis L delivered by a self-emulsifying drug delivery system protects rats from glucocorticoid-induced osteopenia and muscle atrophy. Sci. Rep..

[58] Alghananim A., Özalp Y., Mesut B., Serakinci N., Özsoy Y., Güngör S. (2020). A Solid Ultra Fine Self-Nanoemulsifying Drug Delivery System (S-SNEDDS) of Deferasirox for Improved Solubility: Optimization, Characterization, and In Vitro Cytotoxicity Studies. Pharmaceuticals.

[59] Jianxian C., Saleem K., Ijaz M., Ur-Rehman M., Murtaza G., Asim M.H. (2020). Development and in vitro Evaluation of Gastro-protective Aceclofenac-loaded Self-emulsifying Drug Delivery System. Int. J. Nanomed..

[60] Yeom D.W., Son H.Y., Kim J.H., Kim S.R., Lee S.G., Song S.H., Chae B.R., Choi Y.W. (2016). Development of a solidified self-microemulsifying drug delivery system (S-SMEDDS) for atorvastatin calcium with improved dissolution and bioavailability. Int. J. Pharm..

[61] Janković J., Djekic L., Dobričić V., Primorac M. (2016). Evaluation of critical formulation parameters in design and differentiation of self-microemulsifying drug delivery systems (SMEDDSs) for oral delivery of aciclovir. Int. J. Pharm..

[62] Singh A.K., Chaurasiya A., Singh M., Upadhyay S.C., Mukherjee R., Khar R.K. (2008). Exemestane Loaded Self-Microemulsifying Drug Delivery System (SMEDDS): Development and Optimization. AAPS PharmSciTech.

[63] Ostolska I., Wiśniewska M. (2014). Application of the zeta potential measurements to explanation of colloidal Cr(_2_)O(_3_) stability mechanism in the presence of the ionic polyamino acids. Colloid Polym. Sci..

[64] Hwang C.J., Na Y.G., Huh H.W., Kim M., Lee H.K., Cho C.W. (2019). The Effect of Pharmaceutical Excipients for Applying to Spray-Dried Omega-3 Powder. Appl. Sci..

[65] Tan A., Rao S., Prestidge C.A. (2013). Transforming Lipid-Based Oral Drug Delivery Systems into Solid Dosage Forms: An Overview of Solid Carriers, Physicochemical Properties, and Biopharmaceutical Performance. Pharm. Res..

[66] Katteboina S., Chandrasekhar P., Balaji S. (2009). Approaches for the development of solid self-emulsifying drug delivery systems and dosage forms. Asian J. Pharm. Sci..

[67] Beg S., Katare O.P., Saini S., Garg B., Khurana R.K., Singh B. (2016). Solid self-nanoemulsifying systems of olmesartan medoxomil: Formulation development, micromeritic characterization, in vitro and in vivo evaluation. Powder Technol..

[68] Kasturi M., Agrawal S., Janga K.Y. (2015). Development and Characterization of Ramipril Loaded Solid self Nanoemulsifying Drug Delivery System (SNEDDS) for Improved Oral Delivery of Lipophilic Drugs. Int. J. Pharm. Biol. Sci. Arch..

[69] Bhagwat D.A., Souza J.I.D. (2012). Formulation and evaluation of solid self micro emulsifying drug delivery system using aerosil 200 as solid carrier. Int. Curr. Pharm..

[70] Hauptstein Sabine P.F., Andreas B.S. (2015). Self-nanoemulsifying drug delivery systems as novel approach for pDNA drug delivery. Int. J. Pharm..

[71] Watari A., Hashegawa M., Yagi K., Kondoh M. (2015). Homoharringtonine increases intestinal epithelial permeability by modulating specific claudin isoforms in Caco-2 cell monolayers. Eur. J. Pharm. Biopharm..

[72] Bandivadekar M., Pancholi S., Kaul-Ghanekar R., Choudhari A., Koppikar S. (2013). Single non-ionic surfactant based self-nanoemulsifying drug delivery systems: Formulation, characterization, cytotoxicity and permeability enhancement study. Drug Dev. Ind. Pharm..

[73] Ujhelyi Z., Kalantari A., Vecsernyés M., Róka E., Fenyvesi F., Póka R., Kozma B., Bácskay I. (2015). The enhanced inhibitory effect of different antitumor agents in self-microemulsifying drug delivery systems on human cervical cancer HeLa cells. Molecules.

[74] Zhang Q., He N., Zhang L., Zhu F., Chen Q., Qin Y., Zhang Z., Zhang Q., Wang S., He Q. (2012). The in vitro and in vivo study on self-nanoemulsifying drug delivery system (SNEDDS) based on insulin-phospholipid complex. J. Biomed. Nanotechnol..

[75] Srinivasan B., Kolli A.R., Esch M.B., Abaci H.E., Shuler M.L., Hickman J.J. (2015). TEER measurement techniques for in vitro barrier model systems. J. Lab. Autom..

[76] Mine Y., Zhang J.W. (2003). Surfactants enhance the tight-junction permeability of food allergens in human intestinal epithelial Caco-2 cells. Int. Arch. Allergy Immunol..

[77] Lea T., Verhoeckx K., Cotter P., López-Expósito I., Kleiveland C., Lea T., Mackie A., Requena T., Swiatecka D., Wichers H. (2015). Epithelial Cell Models; General Introduction. The Impact of Food Bioactives on Health: In Vitro and Ex Vivo Models.

[78] USFDA (2006). Guidance for Industry: Dissolution Methods of Azithromycin Pharmaceutical Forms. https://www.accessdata.fda.gov/scripts/cder/dissolution/.

[79] Yeom D.W., Song Y.S., Kim S.R., Lee S.G., Kang M.H., Lee S., Choi Y.W. (2015). Development and optimization of a self-microemulsifying drug delivery system for ator vastatin calcium by using d-optimal mixture design. Int. J. Nanomed..

[80] Sun L., Zhang W., Liu X., Sun J. (2014). Preparation and evaluation of sustained-release azithromycin tablets in vitro and in vivo. Asian J. Pharm. Sci..

[81] Izgelov D., Shmoeli E., Domb A.J., Hoffman A. (2020). The effect of medium chain and long chain triglycerides incorporated in self-nano emulsifying drug delivery systems on oral absorption of cannabinoids in rats. Int. J. Pharm..

[82] Grove M., Pedersen G.P., Nielsen J.L., Müllertz A. (2005). Bioavailability of seocalcitol I: Relating solubility in biorelevant media with oral bioavailability in rats—Effect of medium and long chain triglycerides. J. Pharm. Sci..

[83] Grove M., Müllertz A., Nielsen J.L., Pedersen G.P. (2006). Bioavailability of seocalcitol: II: Development and characterisation of self-microemulsifying drug delivery systems (SMEDDS) for oral administration containing medium and long chain triglycerides. Eur. J. Pharm. Sci..

[84] Patel A.R., Vavia P.R. (2007). Preparation and in vivo evaluation of SMEDDS (Self-microemulsifying drug delivery system) containing fenofibrate. AAPS J..

[85] Bhandari V., Avachat A. (2015). Formulation and characterization of self emulsifing pellets of carvedilol. Braz. J. Pharm..

[86] Behrens D., Fricker R., Bodoky A., Drewe J., Harder F., Heberer M. (1996). Comparison of Cyclosporin A Absorption from LCT and MCT Solutions following Intrajejunal Administration in Conscious Dogs. J. Pharm. Sci..

[87] Porter C.J.H., Kaukonen A.M., Boyd B.J., Edwards G.A., Charman W.N. (2004). Susceptibility to Lipase-Mediated Digestion Reduces the Oral Bioavailability of Danazol After Administration as a Medium-Chain Lipid-Based Microemulsion Formulation. Pharm. Res..

[88] Persson L.C., Porter C.J.H., Charman W.N., Bergström C.A.S. (2013). Computational Prediction of Drug Solubility in Lipid Based Formulation Excipients. Pharm. Res..

[89] Kaukonen A.M., Boyd B.J., Porter C.J., Charman W.N. (2004). Drug solubilization behavior during in vitro digestion of simple triglyceride lipid solution formulations. Pharm. Res..

[90] Kollipara S., Gandhi R.K. (2014). Pharmacokinetic aspects and in vitro–in vivo correlation potential for lipid-based formulations. Acta Pharm. Sin. B.

[91] Zhang X., Wei Y., Cao Z., Xu Y., Lu C., Zhao M., Gou J., Yin T., Zhang Y., He H. (2020). Aprepitant Intravenous Emulsion Based on Ion Pairing/Phospholipid Complex for Improving Physical and Chemical Stability During Thermal Sterilization. AAPS PharmSciTech.

[92] Kalepu S., Manthina M., Padavala V. (2013). Oral lipid-based drug delivery systems—An overview. Acta Pharm. Sin. B.

[93] Pouton C.W. (1997). Formulation of self-emulsifying drug delivery systems. Adv. Drug Deliv. Rev..

[94] Gupta S., Kesarla R., Omri A. (2013). Formulation Strategies to Improve the Bioavailability of Poorly Absorbed Drugs with Special Emphasis on Self-Emulsifying Systems. ISRN Pharm..

[95] Agrawal S., Giri T.K., Tripathi D.K., Alexander A. (2012). A Review on Novel Therapeutic Strategies for the Enhancement of Solubility for Hydrophobic Drugs through Lipid and Surfactant Based Self Micro Emulsifying Drug Delivery System: A Novel Approach. Am. J. Drug Discov. Devel..

[96] Yan B., Ma Y., Guo J., Wang Y. (2020). Self-microemulsifying delivery system for improving bioavailability of water insoluble drugs. J. Nanopart. Res..

[97] Chatterjee B., Hamed Almurisi S., Ahmed Mahdi Dukhan A., Mandal U.K., Sengupta P. (2016). Controversies with self-emulsifying drug delivery system from pharmacokinetic point of view. Drug Deliv..

[98] Akula S., Gurram A.K., Devireddy S.R. (2014). Self-Microemulsifying Drug Delivery Systems: An Attractive Strategy for Enhanced Therapeutic Profile. Int. Sch. Res. Not..

[99] Zhang L., Chen Z., Mao J., Wang S., Zheng Y. (2020). Quantitative evaluation of inclusion homogeneity in composites and the applications. J. Mater. Res. Technol..

[100] Pouton C.W. (1985). Self-emulsifying drug delivery systems: Assessment of the efficiency of emulsification. Int. J. Pharm..

[101] Madan J.R., Patil K., Awasthi R., Dua K. (2019). Formulation and evaluation of solid self-microemulsifying drug delivery system for azilsartan medoxomil. Int. J. Polym. Mater..

[102] Gupta S., Chavhan S., Sawant K.K. (2011). Self-nanoemulsifying drug delivery system for adefovir dipivoxil: Design, characterization, in vitro and ex vivo evaluation. Colloids Surf. A Physicochem. Eng. Asp..

[103] Kohli K., Chopra S., Dhar D., Arora S., Khar R.K. (2010). Self-emulsifying drug delivery systems: An approach to enhance oral bioavailability. Drug Discov. Today.

[104] Buya A.B., Ucakar B., Beloqui A., Memvanga P.B., Préat V. (2020). Design and evaluation of self-nanoemulsifying drug delivery systems (SNEDDSs) for senicapoc. Int. J. Pharm..

[105] Constantinides P.P. (1995). Lipid Microemulsions for Improving Drug Dissolution and Oral Absorption: Physical and Biopharmaceutical Aspects. Pharm. Res..

[106] Azman N.A.Z., Raman I.A., Jantan I., Derawi D. (2019). Formulation Screening of Palm-based Nanoemulsion for an OralDrug Vehicle of Phyllanthin. Malays. J. Chem..

[107] Singh K., Sharma M., Gandhi K. (2012). Recent approaches in self emulsifying drug delivery system. Int. J. Pharm. Sci. Res..

[108] Zaichik S., Steinbring C., Caliskan C., Bernkop-Schnürch A. (2019). Development and in vitro evaluation of a self-emulsifying drug delivery system (SEDDS) for oral vancomycin administration. Int. J. Pharm..

[109] Zhang P., Liu Y., Feng N., Xu J. (2008). Preparation and evaluation of self-microemulsifying drug delivery system of oridonin. Int. J. Pharm..

[110] Parmar B., Patel U., Bhimani B., Sanghavi K., Patel G., Daslaniya D. (2012). SMEDDS: A Dominant Dosage Form Which Improve Bioavailability. Am. J. PharmTech Res..

[111] Almeida S.R.D., Tippavajhala V.K. (2019). A Rundown Through Various Methods Used in the Formulation of Solid Self-Emulsifying Drug Delivery Systems (S-SEDDS). AAPS PharmSciTech.

[112] Mantry S., Majumder D. (2019). Development of Liquid and Solid Self-Emulsifying Drug Delivery System of Silymarin. J. Drug Deliv. Ther..

[113] Dash R.N., Mohammed H., Humaira T., Ramesh D. (2015). Design, optimization and evaluation of glipizide solid self-nanoemulsifying drug delivery for enhanced solubility and dissolution. Saudi Pharm. J..

[114] Kim D.W., Kwon M.S., Yousaf A.M., Balakrishnan P., Park J.H., Kim D.S., Lee B.-J., Park Y.J., Yong C.S., Kim J.O. (2014). Comparison of a solid SMEDDS and solid dispersion for enhanced stability and bioavailability of clopidogrel napadisilate. Carbohydr. Polym..

[115] Cho W., Kim M.-S., Kim J.-S., Park J., Park H.J., Cha K.-H., Park J.-S., Hwang S.-J. (2013). Optimized formulation of solid self-microemulsifying sirolimus delivery systems. Int. J. Nanomed..

[116] Dixit R.P., Nagarsenker M.S. (2008). Self-nanoemulsifying granules of ezetimibe: Design, optimization and evaluation. Eur. J. Pharm. Sci..

[117] Guan Q., Zhang G., Sun S., Fan H., Sun C., Zhang S. (2016). Enhanced Oral Bioavailability of Pueraria Flavones by a Novel Solid Self-microemulsifying Drug Delivery System (SMEDDS) Dropping Pills. Biol. Pharm. Bull..

[118] Man N., Wang Q., Li H., Adu-Frimpong M., Sun C., Zhang K., Yang Q., Wei Q., Ji H., Toreniyazov E. (2019). Improved oral bioavailability of myricitrin by liquid self-microemulsifying drug delivery systems. J. Drug Deliv. Sci. Technol..

[119] Sha X., Wu J., Chen Y., Fang X. (2012). Self-microemulsifying drug-delivery system for improved oral bioavailability of probucol: Preparation and evaluation. Int. J. Nanomed..

[120] Ameeduzzafar, El-Bagory I., Alruwaili N.K., Elkomy M.H., Ahmad J., Afzal M., Ahmad N., Elmowafy M., Alharbi K.S., Md Shoaib A. (2019). Development of novel dapagliflozin loaded solid self-nanoemulsifying oral delivery system: Physiochemical characterization and in vivo antidiabetic activity. J. Drug Deliv. Sci. Technol..

[121] Wei Y., Ye X., Shang X., Peng X., Bao Q., Liu M., Guo M., Li F. (2012). Enhanced oral bioavailability of silybin by a supersaturatable self-emulsifying drug delivery system (S-SEDDS). Colloids Surf. A Physicochem. Eng. Asp..

[122] Mencucci R., Pellegrini-Giampietro D.E., Paladini I., Favuzza E., Menchini U., Scartabelli T. (2013). Azithromycin: Assessment of intrinsic cytotoxic effects on corneal epithelial cell cultures. Clin. Ophthalmol..

[123] Schögler A., Kopf B.S., Edwards M.R., Johnston S.L., Casaulta C., Kieninger E., Jung A., Moeller A., Geiser T., Regamey N. (2015). Novel antiviral properties of azithromycin in cystic fibrosis airway epithelial cells. Eur. Respir. J..

[124] Sullivan D.W., Gad S.C., Julien M. (2014). A review of the nonclinical safety of Transcutol^®^, a highly purified form of diethylene glycol monoethyl ether (DEGEE) used as a pharmaceutical excipient. Food Chem. Toxicol..

[125] Cox S., Sandall A., Smith L., Rossi M., Whelan K. (2020). Food additive emulsifiers: A review of their role in foods, legislation and classifications, presence in food supply, dietary exposure, and safety assessment. Nutr. Rev..

[126] USFDA Center for Drug Evaluation and Research. https://www.accessdata.fda.gov/drugsatfda_docs/nda/2017/209394Orig1s000PharmR.pdf.

[127] Larregieu C.A., Benet L.Z. (2013). Drug discovery and regulatory considerations for improving in silico and in vitro predictions that use Caco-2 as a surrogate for human intestinal permeability measurements. AAPS J..

[128] Shah P., Jogani V., Bagchi T., Misra A. (2006). Role of Caco-2 Cell Monolayers in Prediction of Intestinal Drug Absorption. Biotechnol. Prog..

[129] Li W., Zhou J., Xu Y. (2015). Study of the in vitro cytotoxicity testing of medical devices (Review). Biomed. Rep..

[130] Berridge M.V., Herst P.M., Tan A.S. (2005). Tetrazolium dyes as tools in cell biology: New insights into their cellular reduction. Biotechnol. Annu. Rev..

[131] Tscheik C., Blasig I.E., Winkler L. (2013). Trends in drug delivery through tissue barriers containing tight junctions. Tissue Barriers.

[132] Sha X., Yan G., Wu Y., Li J., Fang X. (2005). Effect of self-microemulsifying drug delivery systems containing Labrasol on tight junctions in Caco-2 cells. Eur. J. Pharm. Sci..

[133] Deli M.A. (2009). Potential use of tight junction modulators to reversibly open membranous barriers and improve drug delivery. BBA-Bioenergetics.

[134] Dimitrijevic D., Shaw A.J., Florence A.T. (2000). Effects of some non-ionic surfactants on transepithelial permeability in Caco-2 cells. J. Pharm. Pharmacol..

[135] Gopalakrishnan S., Durai M., Kitchens K., Tamiz A.P., Somerville R., Ginski M., Paterson B.M., Murray J.A., Verdu E.F., Alkan S.S. (2012). Larazotide acetate regulates epithelial tight junctions in vitro and in vivo. Peptides.

[136] Mariano C., Sasaki H., Brites D., Brito M.A. (2011). A look at tricellulin and its role in tight junction formation and maintenance. Eur. J. Cell Biol..

[137] Liu Y., Chiu G.N.C. (2013). Dual-Functionalized PAMAM Dendrimers with Improved P-Glycoprotein Inhibition and Tight Junction Modulating Effect. Biomacromolecules.

[138] USFDA (2002). Centre for Drug Evaluation and Research Approval Package for: Application Number 50-784. Clinical Pharmacology and Biopharmaceutics Review. www.accessdata.fda.gov.

[139] USFDA (2012). Zithromax^®^ (Azithromycin Tablets and Azithromycin for Oral Suspension). https://www.accessdata.fda.gov/drugsatfda_docs/label/2013/050710s039,050711s036,050784s023lbl.pdf.

[140] Czajkowska-Kosnik A., Szekalska M., Amelian A., Szymanska E., Winnicka K. (2015). Development and Evaluation of Liquid and Solid Self-Emulsifying Drug Delivery Systems for Atorvastatin. Molecules.

[141] Mohd A.B., Sanka K., Bandi S., Diwan P.V., Shastri N. (2015). Solid self-nanoemulsifying drug delivery system (S-SNEDDS) for oral delivery of glimepiride: Development and antidiabetic activity in albino rabbits. Drug Deliv..

